# Insights into Hox Protein Function from a Large Scale Combinatorial Analysis of Protein Domains

**DOI:** 10.1371/journal.pgen.1002302

**Published:** 2011-10-27

**Authors:** Samir Merabet, Isma Litim-Mecheri, Daniel Karlsson, Richa Dixit, Mehdi Saadaoui, Bruno Monier, Christine Brun, Stefan Thor, K. Vijayraghavan, Laurent Perrin, Jacques Pradel, Yacine Graba

**Affiliations:** 1 Institut de Biologie du Développement de Marseille Luminy, IBDML, UMR6216 CNRS, Parc Scientifique de Luminy, Case 907, Marseille, France; 2 Université de la Méditerranée, Parc Scientifique de Luminy, Marseille, France; 3 Department of Clinical and Experimental Medicine, Linkoping University, Linkoping, Sweden; 4 National Centre for Biological Sciences, Tata Institute of Fundamental Research, Bangalore, India; 5 TAGC, U928 Inserm, Parc Scientifique de Luminy, Case 928, Marseille, France; 6 CNRS, Marseille, France; University of California Davis, United States of America

## Abstract

Protein function is encoded within protein sequence and protein domains. However, how protein domains cooperate within a protein to modulate overall activity and how this impacts functional diversification at the molecular and organism levels remains largely unaddressed. Focusing on three domains of the central class *Drosophila* Hox transcription factor AbdominalA (AbdA), we used combinatorial domain mutations and most known AbdA developmental functions as biological readouts to investigate how protein domains collectively shape protein activity. The results uncover redundancy, interactivity, and multifunctionality of protein domains as salient features underlying overall AbdA protein activity, providing means to apprehend functional diversity and accounting for the robustness of Hox-controlled developmental programs. Importantly, the results highlight context-dependency in protein domain usage and interaction, allowing major modifications in domains to be tolerated without general functional loss. The non-pleoitropic effect of domain mutation suggests that protein modification may contribute more broadly to molecular changes underlying morphological diversification during evolution, so far thought to rely largely on modification in gene cis-regulatory sequences.

## Author Summary

Proteins perform essential regulatory functions, including control of gene transcription, a process central to development, evolution, and disease. While protein domains important for protein activity have been identified, how they act together to define the activity of a protein remains poorly explored. The predominant view influenced by prokaryotic transcription factors is that protein domains constitute independent functional modules, required for all aspects of protein activity. In this study, we used Hox proteins, evolutionarily conserved transcription factors playing key roles in the establishment of animal body plans, to examine how protein domains collectively shape protein activity. Results obtained using a broad range of biological readouts highlight a context-dependency in protein domain usage and interaction, revealing that protein domains are non-pleoitropic in nature. This suggests that protein modification may contribute more broadly to molecular changes underlying morphological diversity, so far thought to rely largely on modification of gene cis-regulatory sequences.

## Introduction

How the diversity of animal body plans is established remains a central question in developmental and evolutionary biology [1], [2]. A key step towards understanding the molecular basis underlying diversity is to decipher mechanisms controlling proper genome expression, and how variations in these mechanisms have been at the origin of developmental and evolutionary diversity. While a large number of studies have focused on the impact of cis-regulatory sequences organization (reviewed in [3]), deciphering the intrinsic functional organization of trans-acting transcription factors remains largely unaddressed. Studies have identified functional domains ([4]–[9] and [7], [10], [11] for reviews), but how different protein domains jointly and collectively act for defining the overall activity has been poorly assessed. Yet, a recent study highlights that the synthetic shuffling of protein domains within proteins of the yeast-mating signaling pathway results in the diversification of the mating behavior, demonstrating the importance of protein domain interactions for functional diversification [12].

Hox genes, which encode homeodomain (HD)-containing transcription factors, provide a suitable paradigm to decipher how function is encoded within protein sequence, and how associated changes may constitute the origin of functional specification and diversification. Hox genes have arisen from duplication events of ancestral genes, followed by sequence divergence that promoted the emergence of up to 14 paralogous groups in vertebrates. Hox paralogue proteins display distinct regulatory functions, promoting axial morphological diversification in all bilaterian animals [13]–[17]. Previous work has established that sequence changes in the HD, the DNA binding domain, and a few additional protein domains, have played a major role in the diversification of Hox protein function [4]–[9], [18]–[21]. However, how protein domains functionally interact to shape overall protein activity remains elusive.

**Figure 1:**
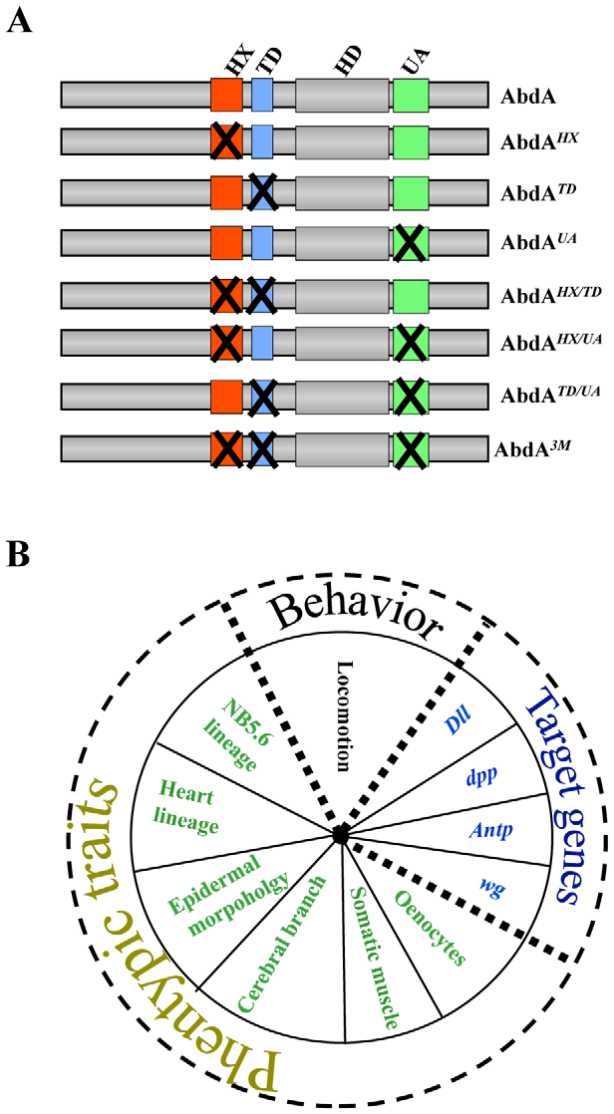
Combinatorial analyses of AbdA protein domains using a variety of biological readouts. A. AbdA protein variants generated. Crosses indicate domain mutations. B. Biological readouts used in this study.

We focused on three protein domains from the *Drosophila* central Hox paralogue protein Abdominal (AbdA, Figure 1). These domains are related by their demonstrated or potential involvement in the recruitment of the Extradenticle (Exd) cofactor, homologous to vertebrate PBX proteins, known to have key roles in establishing Hox functional specificity. The first domain, known as hexapeptide (HX) or PID (Pbx Interacting Domain), with a core YPWM sequence, is found in all Hox paralogue groups, with the exception of some posterior Hox proteins. Biochemical, structural and functional studies have shown that this motif mediates interaction with the Exd/PBX class of Hox cofactors (collectively referred as PBC). The second domain, termed UbdA (UA) is specifically found in the central Hox proteins AbdA and Ultrabithorax (Ubx). This paralogue-specific domain was recently shown to be required for Exd recruitment in the repression of the limb-promoting gene *Distalless* (*Dll*) [8], [22]. The third domain (TD), similar in sequence (TDWM) to the YPWM motif, is also paralogue-specific. The TD motif retains the W that provides strong contact with the PBC class proteins, and matches the sequence of the HX motif in some Hox proteins (eg., Hoxa1). Evidence for an Exd recruiting role of the TD domain in AbdA however remains to be demonstrated.

To start unraveling how protein domains collectively shape Hox protein activity, the effect of single, combined double or triple domain mutations were analyzed using most known AbdA functions as biological readouts. The large functional window covered by the study allows identifying functional attributes of protein domains taken in isolation and collectively, and a quantitative analysis by hierarchical clustering highlights the functional organization of the Hox protein AbdA. Given the phylogeny of the studied protein domains, the work has also implication regarding the mechanisms underlying the evolution of AbdA protein function.

## Results

### Expression of AbdA variants and biological readouts

AbdA variants bearing single or all possible combinations of protein domain mutations (Figure 1A) were ectopically expressed through the binary UAS-Gal4 expression system [23]. Protein levels following induced expression were quantified and experimental conditions ensuring levels close to that of endogenous AbdA were selected (see Materials and Methods). Impact of AbdA variants on target gene control, phenotypic traits and locomotion behavior (Figure 1B), covering AbdA functions of increasing complexity in different tissues, were evaluated in the anterior region where the endogenous AbdA protein is absent. Quantified results (see Text S1) are presented as loss (and in few cases as gain) of regulatory potential.

Eleven functional assays were used to assess domain requirements for AbdA activity (Figure 1B). Four assays rely on the regulation of AbdA target genes, for which evidence of a direct regulation has been previously reported, including the regulation of *Distalless (Dll)* [8], [24], [25] and Antennapedia (*Antp*) [26] in the epidermis, and the regulation of *wingless* (*wg*) [27] and *decapentaplegic* (*dpp*) [28], [29] in the visceral mesoderm. Six assays rely on analysis of phenotypic traits. One of these phenotypic trait, oenocyte specification, results from the regulation of a single target gene [30]. Others, cerebral branch [31], somatic muscles [32], A2 epidermal morphology [33], [34], neuroblast [35], [36] and heart cell lineage specification [37] likely depend of the coordinated regulation of several target genes. Finally, we also used a behavioral trait, larval locomotion, thought to rely on integrated AbdA function in two distinct tissues, the somatic musculature and the nervous system [38].

### Dispensability of the HX, TD, and UA protein domains for somatic muscle specification

**Figure 2:**
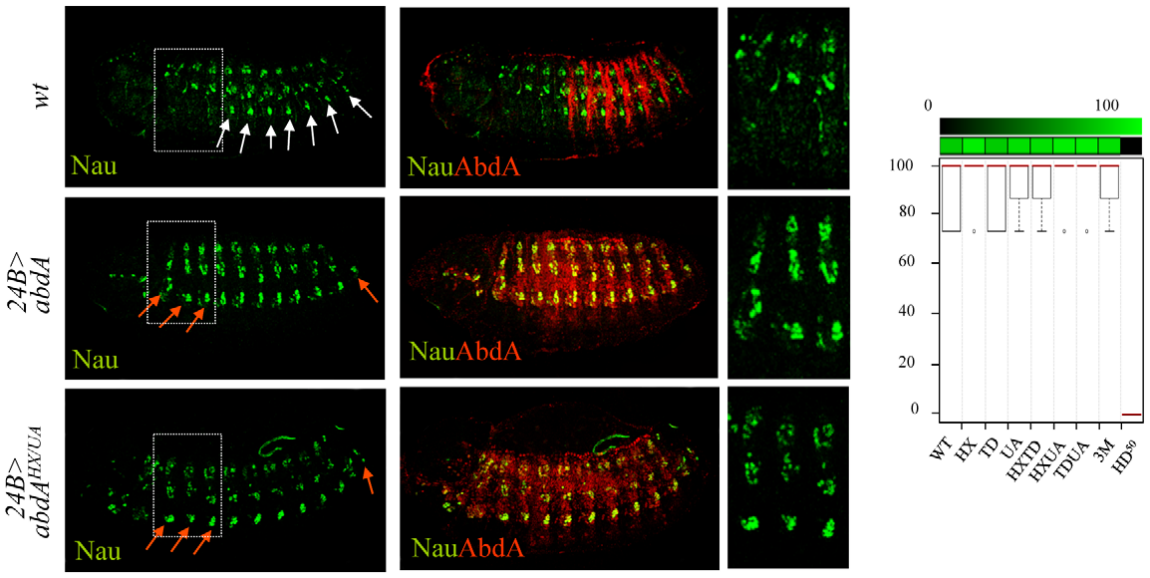
Dispensability of HX, TD, and UA protein domains for somatic muscle specification. Somatic muscles are visualized by Nautilus (Nau, green) expression (upper panels). Expression of AbdA (red) in the thorax using the *24B-Gal4* driver induces abdominal specific muscle pattern (white arrows) in thoracic segments (red arrows) (middle panels). The effect of the AbdA^HX,UA^ variant is illustrated (lower panels). Right panels are magnifications of boxed areas. Graphs (% of remaining activities compared to the wild type AbdA protein (WT) following domain mutations) using the boxplot representation on the right summarize quantitative analyses (see Text S1 and Figure S1 for full illustration). A graded color-coded bar above the graphs illustrates the level of protein activity, ranging from light green (full activity) to black (no activity).

In the somatic musculature, the abdominal specific pattern is characterized by the presence of muscle located ventrally and absent in thoracic segments, a feature that can be visualized by the expression of *nautilus* (*nau*) [32]. This distinction was previously shown to result, at least in part, from the activity of AbdA [32]. Accordingly, anterior ectopic expression of AbdA using the mesodermal driver (*24B-Gal4*) results in ectopic ventral expression of Nau in anterior segments (Figure 2). We found however that none of the AbdA protein domains under study, alone or in combination, was required to specify the abdominal specific features of the somatic musculature (Figure 2 and Figure S1). In the same conditions, a point mutation at position 50 of the homeodomain that impairs AbdA binding to DNA resulted in the loss of Nau inducing capacity (Figure 2 and Figure S1). The dispensability of the HX, TD and UA domains for specifying abdominal features of somatic muscle pattern is consistent with the fact that *nau* activation by AbdA is not dependent upon Exd activity [32], although results below argue that these domains assume other functions than Exd recruitment.

### Single protein domain requirement for NB5–6 CNS lineage specification

**Figure 3:**
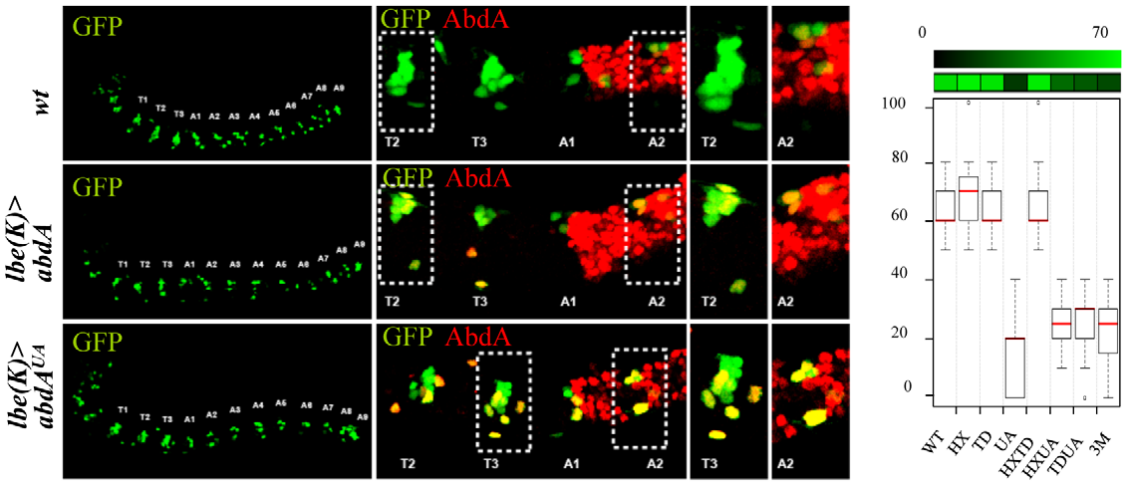
Single protein domain requirement for NB5–6 lineage truncation. Neuroblast 5–6 are visualized using the *ladybird early lbe(K)-Gal4* driver expressing nuclear GFP (green; upper panels). Dotted rectangles highlight differences in cell number of the T2/3 thoracic NB5–6 lineage. Expression of AbdA (red) in NB5–6, through the *lbe(K)-Gal4* driver, leads to thoracic lineage truncation (middle panels). The effect of AbdA^UA^ is illustrated in the lower panel. Graphs (% of remaining activities compared to the wild type AbdA protein (WT) following domain mutations) using the boxplot representation on the right summarize quantitative analysis (see Text S1). A graded color-coded bar above the graphs illustrates the level of protein activity, ranging from light green (full activity) to black (no activity).

In the embryonic central nervous system, a subset of 30 neuroblasts (NB's) found in each hemisegment, including the NB5–6, generate a larger lineage in the thorax than in the abdomen. Recent studies demonstrated that posterior Hox genes, such as *abdA*, impose in the abdomen a smaller NB5–6 lineage by triggering an early cell cycle exit [39]. Misexpression of AbdA within NB5–6 in the thorax using *ladybird(K)-Gal4* result in an early lineage truncation, mimicking the situation that normally occurs in the abdomen, ultimately leading to a smaller thoracic NB5–6 lineage size (Figure 3). Average number of NB5–6 cells in wild type thoracic and abdominal segments was previously estimated at 16 and 6 cells respectively: these values were considered as references for full (100%) or complete loss (0%) of repressive activities of AbdA variants on NB5–6 lineage. Intermediate repressive levels upon ectopic expression with *ladybird(K)-Gal4* were deduced from the quantification of NB5–6 lineage cell numbers in thoracic segments T2/3 (see methods). Results obtained indicate that lineage truncation triggered by AbdA is similarly affected following UA, HX/UA, TD/UA and HX/TD/UA mutations (Figure 3), which can be best explained by a unique requirement of the UA domain for AbdA function.

### Protein domain mutations induces neomorphic activity in the regulation of the *dpp* and *wg* target genes

In the visceral mesoderm, AbdA is expressed in parasegment (PS)8–12. The target genes *wg* and *dpp* are respectively activated (in PS8) and repressed (in PS8–12) by AbdA in the visceral mesoderm. Restricted (PS8) activation of *wg* by AbdA results from the action of the Dpp signal, locally produced by PS7 cells under the control of the Ubx protein [40]. Accordingly, anterior ectopic expression of AbdA only results in a mild activation of *wg*, as activation only occurs in cells experiencing partial repression of *dpp* [27]. Previous work has shown that the HX mutation results in a protein that activates *dpp* instead of repressing it, and consequently more efficiently activates *wg* [41].

**Figure 4:**
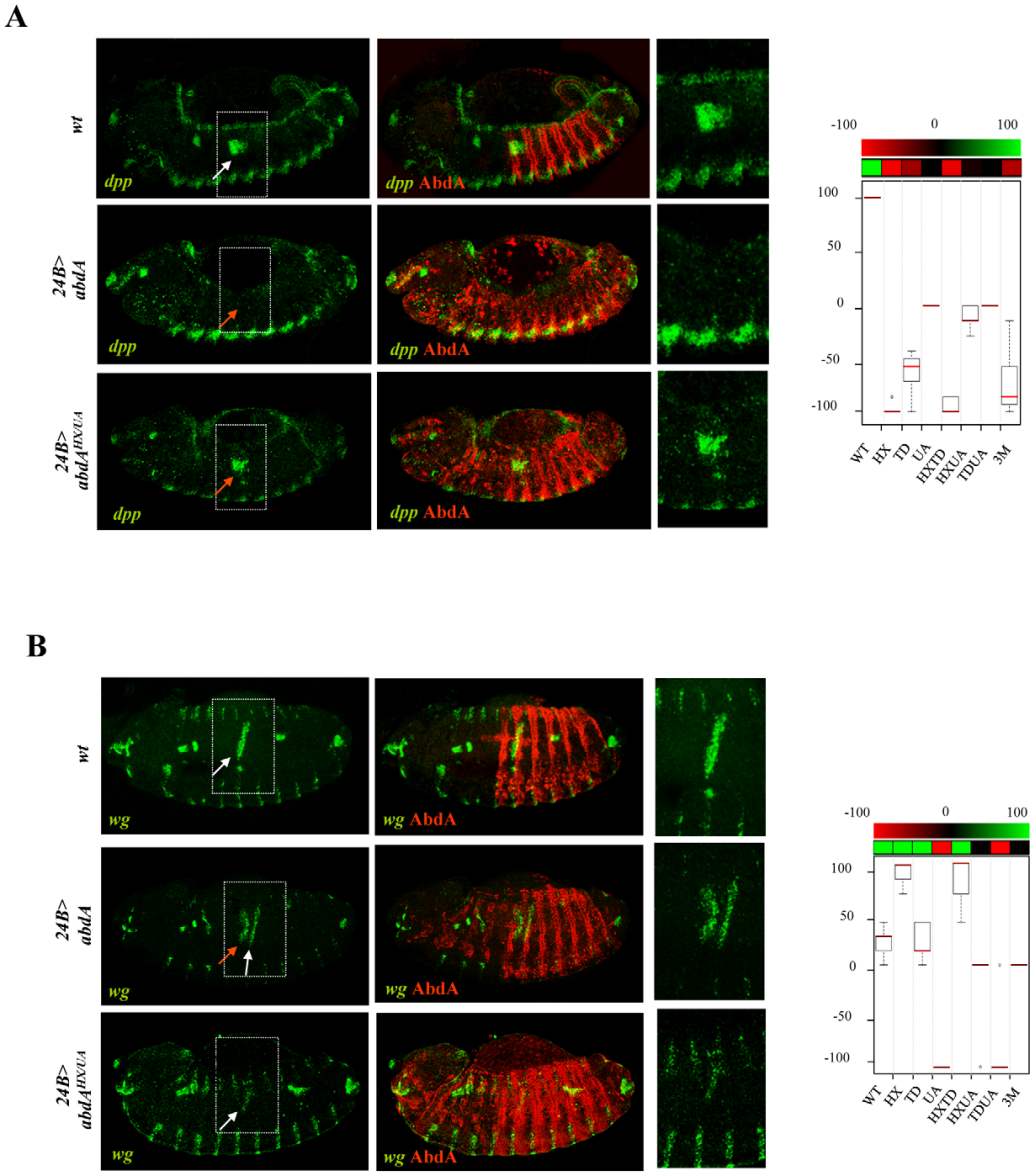
Protein domain mutations inducing neomorphic activities. A. Localized PS7 *dpp* expression (green) in the visceral mesoderm (in situ hybridization, white arrow) relies on posterior repression by AbdA (upper panels). Ubiquitous mesodermal (*24B-Gal4* driven) expression of AbdA (red) represses *dpp* expression in PS7 (red arrow; middle panels) B. Localized PS8 expression of *wg* (green) in the visceral mesoderm (in situ hybridization, white arrow) relies on activation by AbdA. Ubiquitous AbdA in the mesoderm (*24B-Gal4*) induces anterior ectopic *wg* expression (red arrow; middle panels). In A and B, the effect of the AbdA^HX,UA^ variant on *dpp* (A) or *wg* (B) expression is illustrated (lower panels). Right panels are magnifications of the boxed areas. Graphs (% of remaining activities compared to the wild type AbdA protein (WT) following domain mutations) using the boxplot representation on the right summarize quantitative analyses (see Text S1 and Figure S2 (*dpp*) and S3 (*wg*) for full illustration). A graded color-coded bar above the graphs illustrates the level of protein activity, ranging from light green (full activity) to black (no activity). Neomorphic activities, ie qualitative changes in protein activity, are depicted in red.

AbdA variants were expressed with the *24B-Gal4* driver. Levels of regulatory activities were deduced following fluorescent *in situ* hybridization against *dpp* or *wg* in the visceral mesoderm of stage 14 embryos in PS1–PS7, *ie* anterior to endogenous AbdA expressing cells (PS8–12; Figure 4 and Figures S2 and S3). Arbitrary values have been assigned to regulatory activities of AbdA variants. For *dpp* (Figure 4A and Figure S2), no effect on *dpp* expression was scored by 0, normal repression of *dpp* expression in PS7 by 100 (partial repression was never observed) and ectopic activation (instead of repression) of *dpp* was scored by negative values (depending of the number of ectopic sites (see Text S1). For *wg*, in a manner similar to *dpp*, no effect was scored by 0, and positive and negative values were respectively assigned to normal (activation) or abnormal (repression) activities on *wg* expression (Figure 4B and Figure S3; see Text S1).

Results obtained allow two conclusions. First, single domain mutations result in strong modification of AbdA activity. Second, domain mutations often result not only in a quantitative, but also in a qualitative (neomorphic) modification of activity, changing AbdA from an activator to a repressor, or reversely from a repressor to an activator.

### Additive contribution of protein domains for oenocyte specification

Oenocytes form under AbdA control in segments A1–A7. This occurs through AbdA-dependent activation of *Rhomboid* (*Rho*) in a chordotonal organ precursor cell called C1. Expression of *Rho* then enables the secretion of the EGF ligand Spitz that will instruct neighboring epidermal cells to differentiate into oenocytes [30]. In absence of AbdA, the EGF pathway is not locally activated and oenocytes are not specified [30]. Reversely, ectopic expression of AbdA induces oenocytes in thoracic segments.

**Figure 5:**
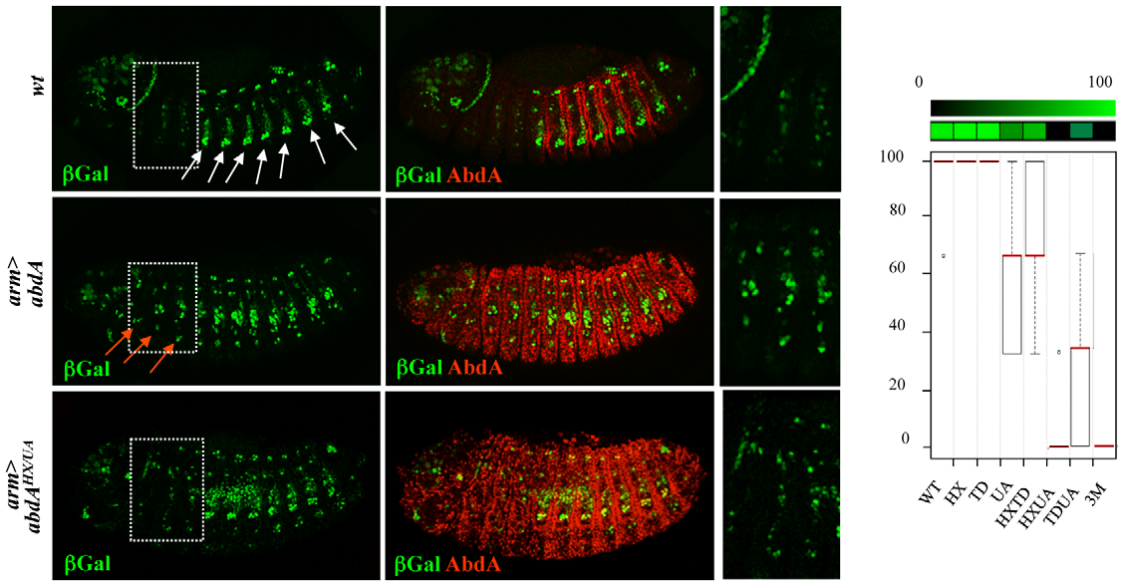
Additive contribution of protein domains. Oenocytes ventrally located in the abdomen are visualized by β-gal (green) driven by the *seven-up* (*svp*) promoter (white arrows, upper panel). Expression of AbdA (red) in the thorax through the *arm-Gal4* driver induces ectopic oenocytes in the thorax (red arrows, middle panels). The effect of the AbdA^HX/UA^ variants is illustrated (lower panels). Boxed areas highlight thoracic segments. Right panels are magnifications of the boxed areas. Graphs (% of remaining activities compared to the wild type AbdA protein (WT) following domain mutations) using the boxplot representation on the right summarize quantitative analyses (see Text S1 and Figure S4 for full illustration). A graded color-coded bar above the graphs illustrates the level of protein activity, ranging from light green (full activity) to black (no activity).

AbdA variants were ubiquitously expressed with the *armadillo (arm)-Gal4* driver. Oenocyte inducing potential of AbdA variants, visualised with the *seven-up (svp)-lacZ* enhancer trap reporter construct, was deduced from the number of thoracic segments that contain ectopic oenocytes (see Text S1). This inductive potential is reduced following single mutations of the UA domain and combined mutation of the HX/TD or TD/UA domains, and is abolished following HX/UA and HX/TD/UA mutations (Figure 5 and Figure S4). These observations suggest an additive contribution of the HX, TD and UA protein domains for oenocyte induction by AbdA, consistent with protein domains acting independently of each other, and contributing uniquely through additive contribution to protein activity.

### Functional redundancy in protein domain usage for trachea and heart lineage specification

**Figure 6:**
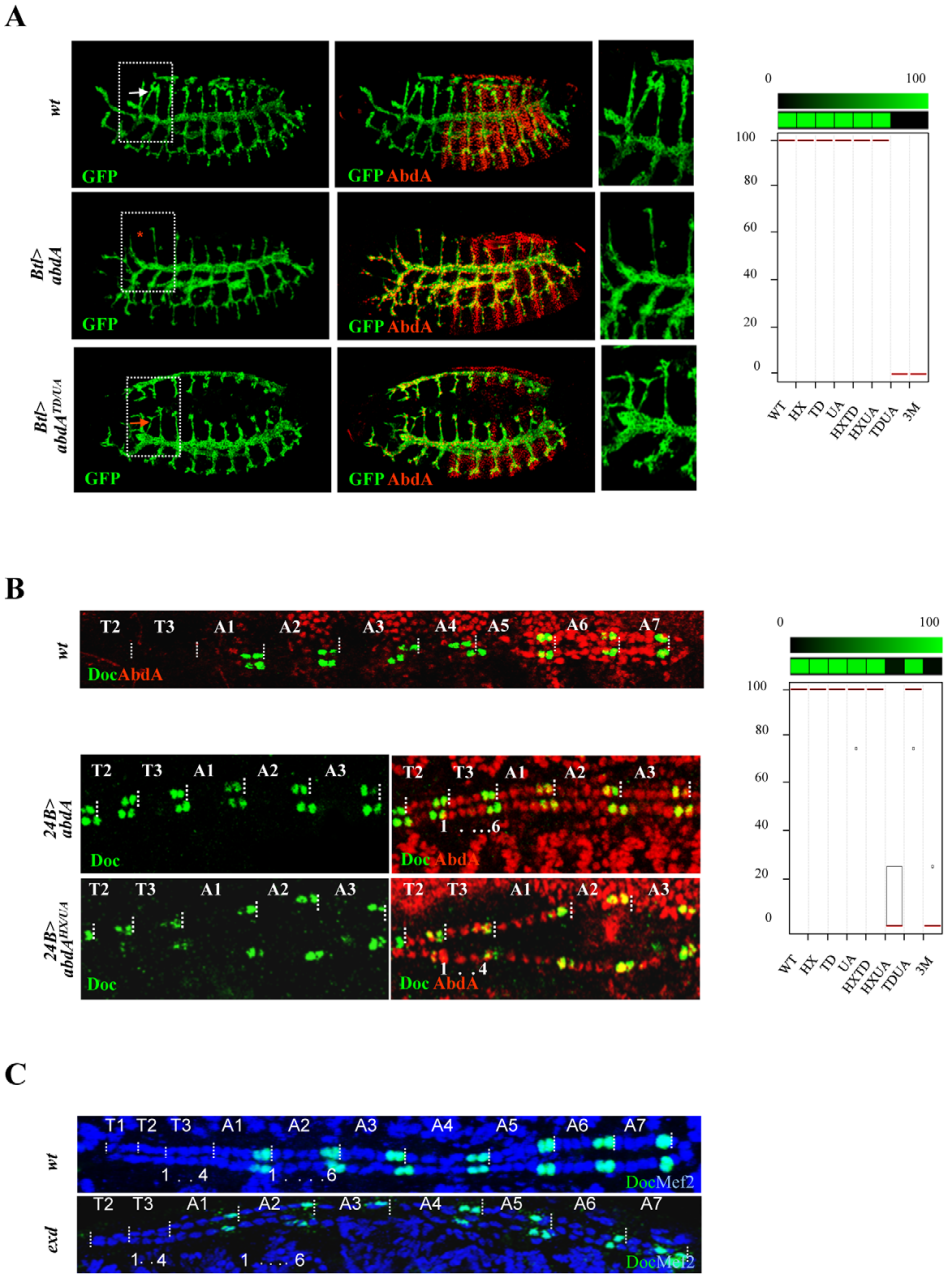
Functional redundancy of the HX, TD, and UA protein domains. A. Tracheal branches are visualized by GFP-driven through the *breathless btl-Gal4* driver (green). The cerebral branch (white arrowhead) forms only in T2 as a result of abdominal repression mediated by AbdA (upper panels). Expression of AbdA (red) in thoracic segments through the *btl-Gal4* driver suppresses cerebral branch formation (red star, middle panels). The effect of the AbdA^TD/UA^ variants is illustrated (arrow, lower panels). Right panels are magnifications of boxed thoracic areas. B. Double immunostaining for AbdA (red) and Doc1 (green) in wild type embryo (upper panel). Magnifications of thoracic segments T2/T3 and abdominal segments A1–3 (middle and lower panels). Expression of AbdA in thoracic segments through the *24B-Gal4* driver promotes a six cell lineage state, with the two anterior most cells expressing Doc1 (middle panels). The effect of the AbdA^HX/UA^ variants is illustrated (lower panels). Graphs in A and B (% of remaining activities compared to the wild type AbdA protein (WT) following domain mutations) using the boxplot representation summarize quantitative analyses (see Text S1 and Figure S5 (cerebral branch) and S6 (heart lineage) for full illustration). A graded color-coded bar above the graphs illustrates the level of protein activity, ranging from light green (full activity) to black (no activity). C. Abdominal hemi-segments in the cardiac tube are composed of six cardiac cells, labeled in blue by Mef2. The two most posterior cells express Doc1 (green). The thoracic hemi-segments lack the anterior Doc1 positive cells. This distinction between thoracic and abdominal segments is not affected following maternal and zygotic loss of *exd*.

The tracheal cerebral branch forms dorsally exclusively in the second thoracic segment T2, in response to repressive activities of Bithorax Hox proteins in T3-A8 segments [42]. This phenotypic trait can be followed by a *breathless* (*btl*) driven GFP reporter that extends posteriorly in the absence of Bithorax complex genes, and that is suppressed in T2 following Btl-driven expression of AbdA in the tracheal system (Figure 6A). Only full repression of cerebral branches was considered and repressive activities of AbdA variants thus correspond to either 0% (no repression) or 100% (full repression) (see Text S1). We found that the repression of the cerebral branch by AbdA is impaired following TD/UA and HX/TD/UA but not HX/UA or HX/TD mutations, revealing a functional redundancy between the TD and UA domains (Figure 6A, and Figure S5).

In the embryonic heart, abdominal segments are made of six pairs of cells, instead of four in thoracic segments [37]. This difference was shown to result from AbdA (and Ubx) promoting the six cell lineage in the abdomen [37], and in the thorax following AbdA ubiquitous expression in the mesoderm driven by the *24B-Gal4* driver ([37], Figure 6B). The visualization of the lineage is facilitated by a Dorsocross (Doc) staining, that labels two cells in each hemisegment, allowing to unambiguously identify each hemisegment. Effects of AbdA variants in cardiac cells specification were visualized by double fluorescent immunostaining against AbdA and Dorsocross (Doc). The six cell lineage inductive capacity of AbdA was scored by counting the number of cardiac cells in the T2 and T3 segments (see Text S1). Results showed that the six cell lineage inductive ability of AbdA is lost following HX/UA and HX/TD/UA mutations (Figure 6B and Figure S6). These observations again highlight functional redundancy, but between the UA and HX domains, instead of TD and UA domain as observed in cerebral branch specification. Additional examples of functional redundancy, yet in more complex pattern of interactions between protein domains were found in the biological contexts described below.

### Mutually suppressive interaction of protein domains in the regulation of the *Dll* and *Antp* direct target genes, the specification of epidermal morphology, and larval locomotion

**Figure 7:**
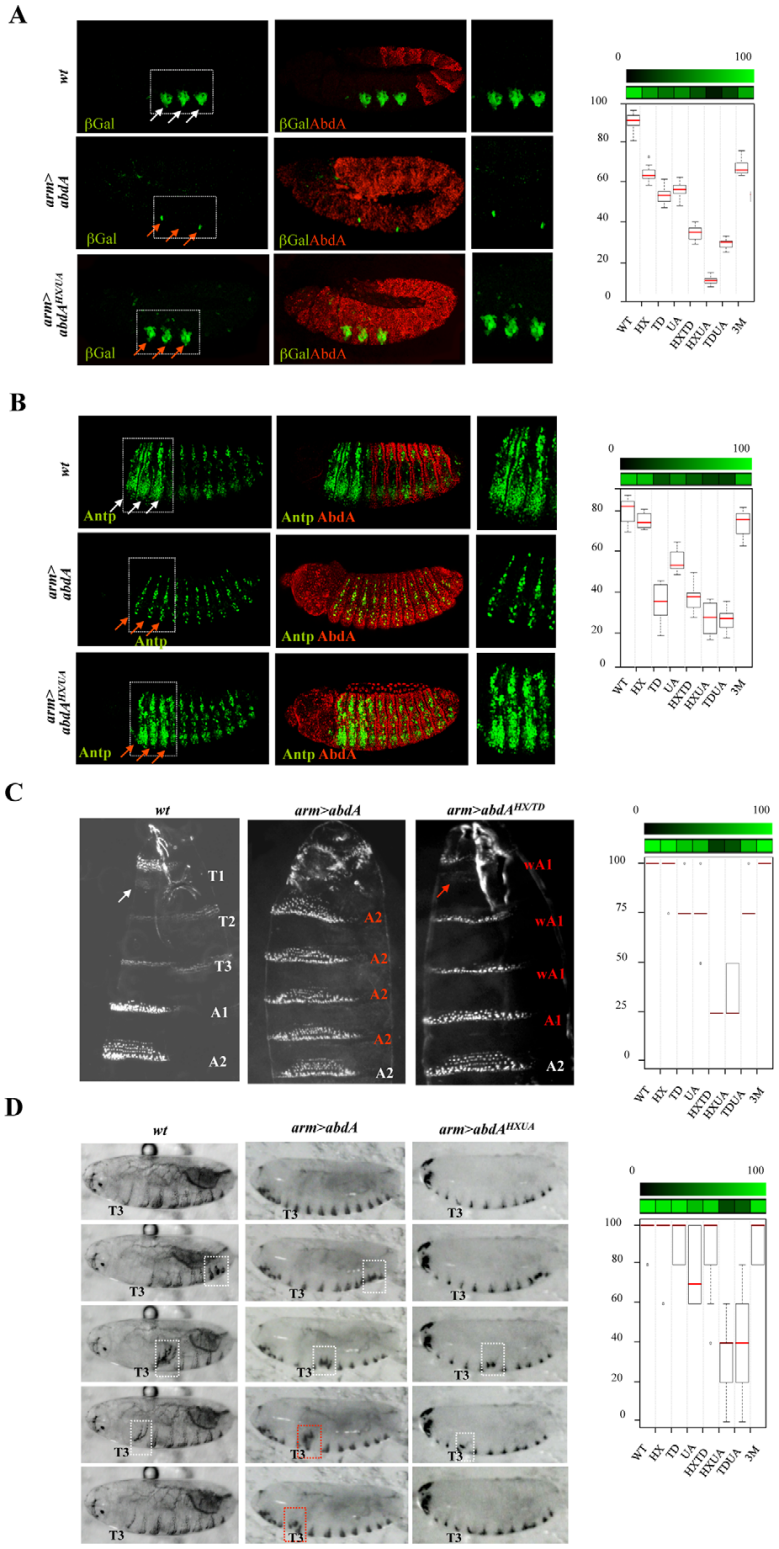
Mutually suppressive interaction of protein domains. A. Thoracic restricted expression of *Dll* (white arrows) followed by *Dll* enhancer driven β-gal (green) results from repression by AbdA (red) in the abdomen (upper panel). Ubiquitous AbdA expression driven by *arm-Gal4* represses *Dll* thoracic expression (red arrows, middle panels). The effect of the AbdA^HX/UA^ variants is illustrated (lower panels). Right panels are magnification of boxed thoracic areas. B. Increased thoracic Antp expression (green, white arrows) results from AbdA (red) repression in the abdomen (upper panels). Ubiquitous AbdA expression driven by *arm-Gal4* represses Antp expression in the thorax (red arrows, middle panels). The effect of the AbdA^HX/UA^ variants is illustrated (lower panels). Right panels are magnification of boxed thoracic areas. C. Abdominal segments are characterized by refringent denticles organized in a trapezoidal shape in segments A2 but not A1, while T2/T3 thoracic segments harbors thinner denticles (left panel). Upon AbdA thoracic expression driven by *arm-Gal4*, the first abdominal segment A1 and thoracic segments acquire abdominal features, including abdominal type of denticles, trapezoidal organization of denticles and suppression of a T1 specific feature (white arrow), the “beard” (middle panel). Full or intermediate transformations were observed for AbdA variants (see Text S1 for quantifying criteria). The effect of the AbdA^HX/TD^ variants is illustrated (right panel). Weak A1 (wA1) stands for a transformation of thoracic denticles toward abdominal type of denticles, with an organization typical of A1, but with only a partial suppression of the beard in T1 (arrow). D. Snapshots from movies illustrating locomotion in wild type larvae (left panels), or in larvae expressing ubiquitously AbdA (middle panels) or AbdA^HX/UA^ variant (right panels) driven by the *arm-Gal4* driver. White boxed areas show the progression of a peristaltic waves in the abdomen. The red boxed area shows an ectopic peristaltic wave in the thorax following ectopic AbdA expression in the thorax. Graphs in A–D (% of remaining activities compared to the wild type AbdA protein (WT) following domain mutations) using the boxplot representation summarize quantitative analyses (see Text S1 and Figure S7 (*Dll*), S8 (Antp), and S9 (A2 epidermal morphology) for full illustration, and Figure S10 for data on larval locomotion experiments. A graded color-coded bar above the graphs illustrates the level of protein activity, ranging from light green (full activity) to black (no activity).

The limb-promoting gene *Distalles* (*Dll*) and Hox gene *Antennapedia* (*Antp*) are direct targets of AbdA [26], [43]. The ability of AbdA variants, following ubiquitous expression through the *arm-Gal4* driver, to repress *Dll* (Figure 7A and Figure S7) and *Antp* (Figure 7B and Figure S8) was evaluated by examining the activity of a Hox responsive *Dll* enhancer (DME, [44]) and the expression of the Antp protein, respectively (see Text S1). Single domain mutations do not strongly affect repressive activities of AbdA on *Dll* and *Antp*, leading to a mean loss of 40%, with the exception of the TD mutation, which affects more (60%) the repressive activities on *Antp*. Combining domain mutations leads to stronger effects: in the case of *Dll*, simultaneous mutation of the HX and UA domains almost completely abolishes AbdA repressive activities, while in the case of *Antp* simultaneous mutation of the HX and UA domains or TD and UA domains results in a loss of 70% of AbdA repressive activity. More surprisingly, simultaneous mutation of the HX, TD and UA domains does not compromise further AbdA activity but instead restores a significant level of repressive activity, comparable to that of single domain mutated AbdA variants. This indicates that the three protein domains do not provide independent regulatory input, but likely act in interactive and mutually inhibitory ways.

A similar yet more complex pattern of domain interactions was observed in the specification of A2 epidermal morphology. In this tissue, AbdA promotes the formation of a stereotyped trapezoidal arrangement of denticle belts (Figure 7C). The potential of AbdA variants to specify A2 epidermal morphology was assessed following *arm-Gal4* driven expression by scoring the denticle belts morphology and organisation in transformed A1 and thoracic segments (Figure 7C and Figure S9). Epidermal specification was not impaired by HX and slightly reduced by UA or TD mutations. Simultaneous mutation in two domains suggests functional redundancy between HX and TD, UA and HX but not between UA and TD domains. As noticed previously for the regulation of *Dll* and *Antp* in the epidermis, mutating the three domains simultaneously restores the activity, generating a protein that displays an activity close to the wild type protein.

In many animals including vertebrates, locomotion results from the coordinated action of regionally distinct sets of movements. *Drosophila* larvae crawl by means of three region specific movements [38]. The locomotion cycle starts by a contraction of the most posterior abdominal segments (A8/A9), followed by a wave of peristaltic movement in A1–A7, where each segment is transiently lifted up (D/V movement), pulled forward and lowered, starting from A7. When the wave reaches A1, the thoracic and head segments start moving by a telescopic type of movement (A/P movement), occurring through contraction of anterior segments [38]. It was established that AbdA is necessary and sufficient to specify the abdominal type of movement, namely abdominal peristalsis [38]. The potential of wild type and AbdA variants to promote abdominal peristalsis was evaluated following *arm-Gal4* driven expression (Figure 7B), by scoring in the T3 thoracic segment D/V movements (see Text S1). Single domain mutations do not significantly alter promotion of abdominal peristalsis (Figure 7D and Figure S10). Again, two types of functional redundancy were observed: between the TD and UA domains, and to a lesser extent between the HX and UA domains. As in the case of *Dll* and *Antp* regulation and A2 epidermal morphology specification, triple domain mutation corrected the effects of double mutations, with a protein promoting abdominal peristalsis as efficiently as the wild type protein, providing an additional example of mutually suppressive activity of protein domains.

### Multifunctionality of protein domains revealed by Exd-dependency

Previous studies have established that Exd is required for *Dll* [25] and *wg* [45] regulation, oenocytes [30] and epidermal morphology specification [46], and neuroblast lineage commitment [37], while dispensable for *Antp* [46] and *dpp* [47] regulation. In the case of cerebral branch specification, no conclusion could be reached since loss of Exd results in the absence of cerebral branch formation in the T2 segment [48]: this positive input of Exd hinders the assessment of a possible contribution for AbdA mediated cerebral branch repression in abdominal segments.

The potential implication of Exd in AbdA-mediated heart lineage commitment and larval locomotion is not known. Staining for Doc1 in embryos deprived for maternal and zygotic Exd showed that the abdominal hemi segments adopt the AbdA-dependent six cell lineage, showing the dispensability of Exd for this AbdA function (Figure 6C). The requirement of Exd for larval locomotion has been examined in *homothorax* (*hth*) mutant that impairs Exd nuclear transport and mimics *exd* maternal and zygotic loss [49]. The absence of peristaltic waves in this genetic context indicates a strict requirement of Exd for abdominal peristalsis (Figure S10).

Taken together with the protein domain requirement results, the *exd* dependency indicates that the HX, UA and TD domains, known (HX and UA) or candidate (TD) Exd recruiting domains, are also required for Exd-independent function. This is supported by the HX/UA requirement for heart lineage specification, by the HX and UA requirement for proper regulation of the *dpp* target gene, the HX/TD requirement for *Antp* repression and the requirement of TD for *dpp* target regulation. Collectively, this highlights that the HX and UA (and likely TD) protein domains are multifunctional, serving in some biological context Exd interaction function, while in others, they are used differently, for a molecular activity that still remains to be defined.

### Hierarchical clustering reveals two functional modules and a predominant role for the UA domain

**Figure 8:**
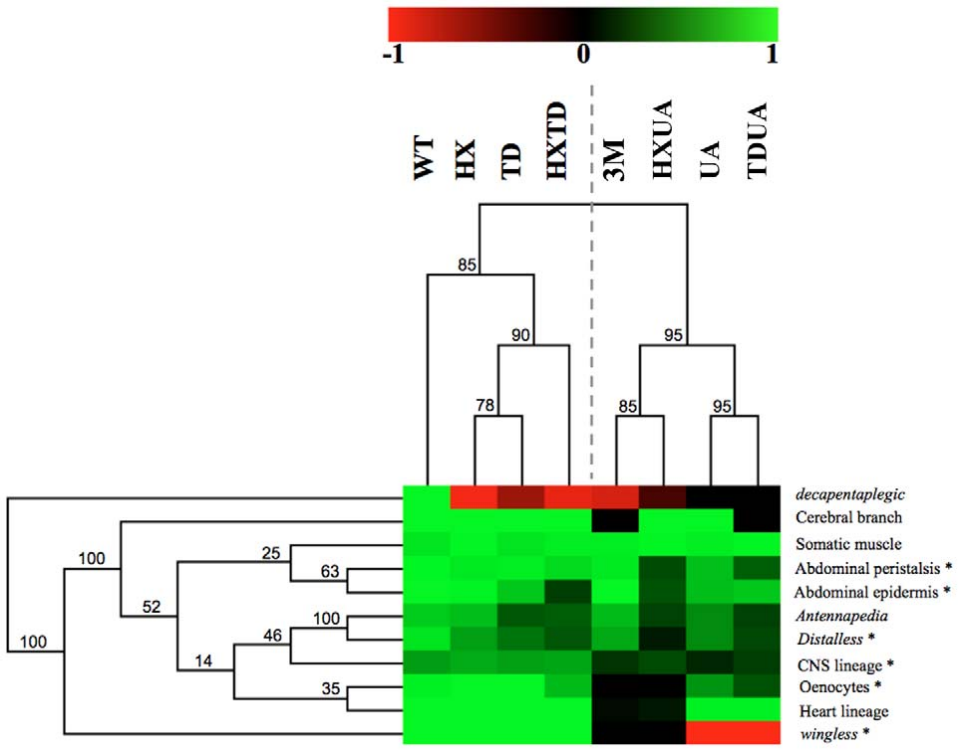
Hierarchical clustering of AbdA domain requirements. Hierarchical clustering of domain contribution reveals two functional modules and a predominant role for the UA domain. A graded color-coded bar above the graphs illustrates the level of protein activity, ranging from light green (full activity) to black (no activity). Neomorphic activity is depicted in red. Asterisks indicate Exd dependent biological readouts. Jacknife values are indicated for each node.

The complete set of quantitative data was analyzed using a hierarchical clustering method (Figure 8; see Materials and Methods). Clustering according to biological readouts does not reveal any clear grouping, regarding for instance developmental stage or tissue type, suggesting that the forces that govern domain usage and interaction between protein domains mostly reside in the regulated target gene. By contrast, clustering according to protein domains clearly reveals a hierarchical requirement of the domains for the various AbdA functions analyzed here. A bipartition of AbdA variants is observed, with the mutants for the HX, the TD and HX/TD domains on the one hand, and variants mutant for the UA domain, alone or in combination, on the other hand. Such bipartition suggests the existence of two functional modules that can be distinguished based on UA domain requirement. The first module, which relies mostly on the HX and TD domains, is used for a small subset of AbdA functions only. The second module relies on the activity of the HX, TD and UA domains, yet the requirements of the HX and TD domains are revealed only in UA deficient context. Thus, the driving force in this second functional module is the UA domain, as its mutation unmasks the requirement for the HX and TD domains, which is not revealed by their single or combined mutations. These results identify a prominent role of the UA domain in AbdA function.

## Discussion

### A different complementary approach to Hox protein function

Studies towards deciphering the mode of action of Hox proteins have so far essentially concentrated on how individual protein domains contribute to protein function. These focused approaches allowed in depth analyses, unraveling the intimate molecular and sometimes structural details of how protein domains contribute to protein function, providing decisive insights into how Hox proteins reach specificity. This work provides a different complementary approach towards deciphering the mode of action of Hox proteins. First it aims at studying protein domains in combinations, using combined and not only single protein domain mutations, considering that the overall protein activity is likely not a sum of the activity of individual protein domains, and that novel properties may emerge from interactions between protein domains. Second, it uses extensive in vivo biological readout, (most of the known AbdA functions), instead of a single or a few functions. While impairing the in depth analyses of previous focused approaches, the large functional window covered by this study allows the identification of features underlying the intrinsic functional organization of the Hox protein AbdA.

Although the approach taken relies on a gain of function strategy, special care was taken to select experimental conditions where proteins were expressed closed to physiological levels of expression. Biological readouts considered are functions that AbdA can sustain in ectopic places, suggesting that availability of AbdA protein partners is not a limitation of the experimental strategy chosen. Finally, the effects of expressing the AbdA variants (in all eleven biological readouts) were scored in regions anterior to the endogenous AbdA expression domain (*ie* in cells where the endogenous wild type gene product is not present), avoiding any further complexity that may result from competition with the endogenous AbdA protein.

Below, we summarize how results obtained shed light on the mode of action of the Hox protein AbdA and discuss the evolutionary implications.

### Functional attributes and mode of protein domain usage: Implication for robustness and diversity

This study identifies salient features underlying the intrinsic functional organization of the AbdA Hox transcription factor. Protein domains often display functional redundancy, with strong effects in most cases requiring simultaneous mutations of two or three domains. Redundancy was frequently observed between the HX and UA domains, or between the TD and UA domains, while redundancy between the HX and TD domains is less frequent (Figure 8). This indicates that redundancy does not necessarily rely on functional compensation through structurally related domains, since the HX and TD are closely related domains, while the UA domain is completely unrelated. Thus, functional redundancy rather reflects the potential to perform similar activities through distinct molecular strategies. This property likely confers robustness to Hox protein activity, accommodating mutations in protein domains without generally impacting on regulatory activities.

Protein domains within AbdA also generally do not act as independent functional modules, but instead display a high degree of interactivity, as demonstrated by the non-additive effects of domain mutations in the majority of the biological readouts studied. In addition, protein domains are often multifunctional, in the sense that they serve different molecular functions. This is illustrated by the fact that the HX and UA domains, previously described to mediate Exd recruitment, are also required for Exd-independent processes. Thus domain interactivity and multifunctionality are hallmarks of AbdA regulatory activity. These properties provide means to apprehend the bases underlying Hox functional diversity with a restricted number of functional modules, and therefore may account for the variety of Hox-controlled biological functions.

Protein domain usage and interaction between protein domains in AbdA strongly depends on the biological readout, suggesting that domain usage largely depends on the regulated target gene, and hence on the identity of the gene cis regulatory sequences. Recent reports support that DNA sequences impact on Hox protein activity: Hox binding site neighboring sequences are important for proper regulation of the *reaper* downstream target [50]; Sex combs reduced changes its conformation and activity depending on the cognate sequence [51]. Of note, a role for the target sequence in controlling the structure and activity of the glucocorticoid receptor has also been recently reported [52], indicating that this may generally apply for many DNA binding transcription factors.

### Mechanisms underlying the evolution of protein function

**Figure 9:**
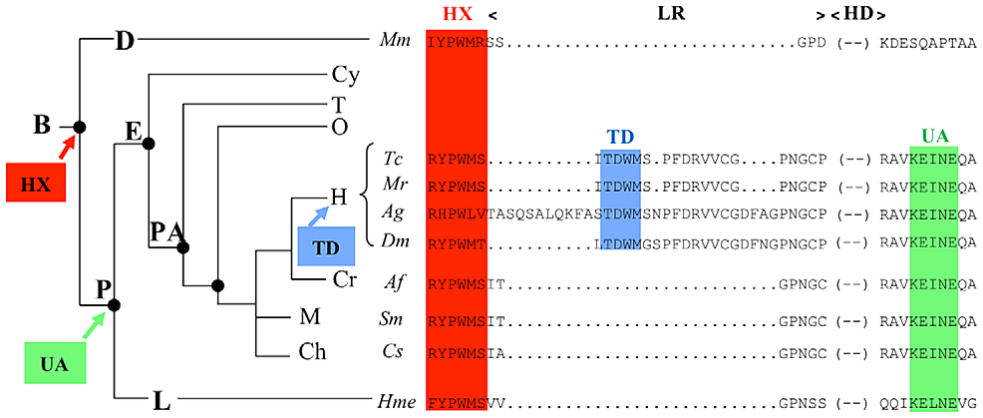
Phylogeny of the HX, TD, and UbdA protein domains. Abbreviations are as follows: B: bilaterians; P: protostomes; D: deuterostomes; L: lophotrochozoa; E:ecdysozoa; Cy, cycloneuralia. PA: pan-arthopods; O: onycophores; T: tardigrades; Ch: chelicerates; M; myriapods; Cr: crustaceans; H:hexapods. Sequence alignment spanning the HX, TD and UA domains are shown for representatives of the four main arthropod branches (*Tc: Tribolium castaneum*; *Mr: Myrmica rubra*; *Ag: Anophela gambiae*; *Dm: Drosophila melanogaster*) and for a representative of deuterostomes (*Mus musculus*, *Mm*) and lophotrochozoa (*Hirudo medicinalis*, *Hme*).

Our results also have implication on how modifications in protein sequences are translated into changes in protein function during evolution. The HX domain, common to all Hox proteins, is ancient and found in all bilaterians, and provides a generic mode of PBC interaction (Figure 9). The UA domain, specific to some central Hox proteins (AbdA and Ubx in *Drosophila*), was acquired later, at the time of protostome/deuterostome radiation. It provides a distinct yet to be characterised PBC interaction mode, specific to some Hox paralogues only, allowing fine-tuning of Hox protein activity [22]. TD is found only in insect AbdA and not in Ubx proteins, suggesting that it arose after the duplication that generated Ubx and AbdA in the common ancestor of insects (Figure 9). Remarkably, within AbdA arthropod proteins, the HX domain has significantly diverged in some lineages like anopheles, while the TD domain has been strictly conserved.

Conceptually, two non-exclusive models could account for the evolution of protein function following the acquisition of a novel protein domain. In the first one, the acquisition provides a novel molecular and functional property, which adds to pre-existing ones. This is for example the case for the acquisition of the QA domain that confers repressive function to Ubx [6], and the acquisition/loss of HX or LRALLT domains by Futzitarazu (Ftz) from distinct insect species, which provides Ftz with the capacity to recruit either Exd or FtzF1 cofactors and switches its activity from a Hox to a segmentation protein [53]. In the second model, the acquisition of a novel protein domain interferes with the activity of pre existing domains, reorganizing the intrinsic functional organization of the protein. This view is supported by the predominant role of the UA domain and the widespread domain interactivity seen in this study.

### More room for changes in protein activity during morphological evolution

Evolutionary changes in animal morphology is thought to mostly rely on changes in cis-regulatory sequences [1]. This is conceptually supported by the modular organization of cis-regulatory sequences, allowing subtle and cell specific changes in gene expression not deleterious for the animal. Experimentally, it is largely supported by the correlation between expression of key developmental regulatory genes and morphological changes (for example see [54]), and by changes in cis-regulatory sequences that impact on morphological traits [55]–[59]. Changes in animal morphology could also result from changes in protein sequence and function, as shown for Hox proteins in the morphological diversification in arthropods [4], [6]. However, changes in protein function are not believed to broadly contribute to morphological diversification during animal evolution, based on the assumption that changes in protein sequences are expected to have pleiotropic effects, which as such, do not provide a mean to convey subtle and viable evolutionary changes.

Our work grasps redundancy and selectivity in protein domain usage and as salient features of AbdA transcription factor intrinsic regulatory logic: even the HX domain, evolutionarily conserved in all Hox proteins, is essential for only one AbdA function, and often acts in a redundant way with the TD or the UA protein domains. Selective use of protein domains is also supported by findings of a few smaller scale studies of three other *Drosophila* Hox proteins: viable missense or small deletion mutations within the Scr protein coding sequences falls in different allelic series when examined for three distinct biological readouts [60]; deletion of C-terminal sequences of the Ubx protein, starting from an insect specific QA protein domain preferentially affects a subset of Ubx function [61]; dispensability of the HX was reported for the leg inducing capabilities of the Antp Hox protein, while required for other Antp functions [62]. This context dependent selective mode of protein domain usage, or differential pleiotropy, may be essential for the evolution of Hox protein functions, as it ensures developmental robustness of a Hox-controlled program while being permissive to evolutionary changes endowing novel functions to preexisting protein domains. In addition, our work also establishes that interactivity between protein domains is highly context dependent, suggesting that Hox protein function not only relies on selective mode of protein domain usage but also on selective mode of protein domain interactivity. Altogether, these observations challenge the view that changes in protein sequences necessarily have pleiotropic effects, giving more room for protein changes in the evolution of animal body plans.

## Materials and Methods

### Flies, egg collections, cuticle preparations, *in situ* hybridization, and immunostaining

*24B-Gal4* and *arm-Gal4* were used as embryonic mesodermal and ubiquitous drivers, respectively. *Btl-Gal4* and *lbe(K)-Gal4* for specific expression in the tracheal system and NB5–6 neuroblasts, respectively. The *DME-lacZ* and *svp-lacZ* lines are respectively from R. Mann (Columbia Univ., NY, USA) and S. Zaffran (IBDML, Marseille, France). *exd^XP11^* and *hth^P2^* alleles were used. Embryo collections, cuticle preparations, *in situ* hybridizations, and immunodetections were performed according to standard procedures. Digoxigenin RNA-labelled probes were generated according to the manufacturer's protocol (Boehringer Mannheim, Gaithersburg, MD) from *wg* and *dpp* cDNAs cloned in Bluescript. Primary antibodies used are: anti-Antp (4C3, dilution 1/100, Developmental Studies Hybridoma Bank (DSHB)); rabbit anti-AbdA (1/1000); guinea-pig anti-Doc2+3 (1/400) and rabbit anti-Dmef2 (1/2000) from L. Perrin (IBDML, Marseille, France); rabbit anti-Exd (1/1000) from R. Mann; rabbit anti-Nau (1/100) from BM Paterson (University of Texas Southwestern Medical Center, Dallas, TX); rabbit (1/500) or mouse (1/200) anti-GFP (1/500) from Molecular Probes; chicken anti-GFP (1/1000) from Aves labs; mouse anti-β-galactosidase (1/1000) from Promega; rabbit anti-β-galactosidase (1/1000) from MP Biomedical; anti-digoxigenin coupled to biotin (1/500) from Jackson. Secondary antibodies coupled to Alexa 488, Alexa 555 (Molecular Probes) or to biotin (Jackson) were used at a 1/500 dilution.

### Constructs, transgenic lines, biological readouts, and quantification procedures

AbdA variant were generated by PCR. Domain mutations were YPWM→AAAA; TDWM→AVAI; KEINE→KAAAA. The homeodomain point mutation alleviating DNA binding is a mutation of position 50 (Q→K; [47]). Constructs were cloned in pUAST or pUASTattB vectors for transgenic line establishment. Lines were crossed with the appropriate driver, and collected embryos were stained with anti-AbdA to select the conditions (line and temperature) that result in expression levels similar (+/−15%) to AbdA wild type levels in A2 (see [22] for a detailed description of the procedure). Procedures used for quantification of biological readouts using at least 10 embryos of each genotype are provided in Text S1.

### Hierarchical clustering of domain requirements

A matrix containing the values corresponding to the readout was built. The extreme values were given to the total loss of activity (value 0), and to the wild type activity (value 1 for 100% of activity). A hierarchical clustering algorithm (with Euclidian distance and average linking) was applied to the matrix using the MeV software suite [63]. The jacknife method was used for re-sampling the data and provides a statistical support for each tree node.

### Boxplot data representation

Boxplots drawn using the R-Software. Boxplot depicts the value distribution obtained for each tested genotype. Black points correspond to individual counts.

## Supporting Information

Figure S1 (Full data for Figure 2.) AbdA protein domain requirements for somatic muscle specification. Somatic muscle cells are visualized by Nau immunostaining (green). A representative embryo is shown for each AbdA variant, as indicated. AbdA variants were ubiquitously expressed (red) in the mesoderm with the *24B-Gal4* driver. Dotted white rectangles highlights segments (thoracic or abdominal segment A8) where the effect of AbdA variants was determined.
(PDF)

Figure S2 (Full data for Figure 4A.) AbdA protein domain requirements for the regulation of the *dpp* direct target gene. The regulatory effect of AbdA variants on *dpp* expression was determined by in situ hybridisation to *dpp* transcripts (green). Arrow indicates the expression of *dpp* in PS7 of the visceral mesoderm. Gain of *dpp* expression in the visceral mesoderm is indicated by white dots, while loss of PS7 expression is denoted by the absence of arrow. AbdA variants were ubiquitously expressed (red) in the mesoderm with the *24B-Gal4* driver. A representative embryo is shown for each AbdA variant.
(PDF)

Figure S3 (Full data for Figure 4B.) AbdA protein domain requirements for the regulation of the *wg* direct target gene. The regulatory effect of AbdA variants on *wg* expression was determined by in situ hybridisation to *wg* transcripts (green). Arrow indicates the expression of *wg* in PS8 of the visceral mesoderm. Gain of *wg* expression in the visceral mesoderm is indicated by white dots, while loss of PS8 expression is denoted by the absence of arrow. Restricted PS8 activation of *wg* by AbdA results from the action of the Dpp signal, locally produced by PS7 cells under the control of the Ubx protein [40]. Accordingly, anterior ectopic expression of AbdA only results in a mild activation of *wg*, as activation only occurs in cells experiencing partial repression of *dpp* [27]. Previous work showed that the HX mutation results in a protein that activates *dpp* instead of repressing it, and consequently more efficiently activates *wg* [41].
(PDF)

Figure S4 (Full data for Figure 5.) AbdA protein domain requirements for oenocytes specification. Oenocytes, restricted to A1–A7 abdominal segments, were marked using a *seven-up* svp-lacZ construct (β-galactosidase staining in green). Ubiquitous expression of AbdA variants (red) with *arm-Gal4* induces the formation of ectopic oenocytes in thoracic segments. A representative embryo is shown for each AbdA variant. Boxed areas highlight thoracic segments.
(PDF)

Figure S5 (Full data for Figure 6A.) AbdA protein domain requirements for cerebral branch specification. The *breathless btl-Gal4* driver, specific to tracheal branches, was used to simultaneously express the AbdA variants (red) and the GFP reporter protein (green), allowing visualisation of tracheal defects. Presence (white arrow) or absence (red star) of the cerebral branch following ectopic expression of the AbdA variants is shown.
(PDF)

Figure S6 (Full data for Figure 6B.) AbdA protein domain requirements for the specification of heart cells. Thoracic segments are formed of four pairs of cardiac cells, while abdominal ones are composed of six pairs of cardiac cells. The two supplementary pairs of abdominal cardiac cells express Doc1 (green). Ectopic expression of AbdA (red) in the mesoderm driven with the *24B-Gal4* driver induces additional Doc1-expressing cells in thoracic segments that are now composed of six pairs of cells. A representative embryo is shown for each AbdA variant.
(PDF)

Figure S7 (Full data for Figure 7A.) AbdA protein domain requirements for the regulation of the *Dll* direct target gene. The regulatory effect of AbdA variants (red) on *Dll* expression was determined by the activity of the *Dll* DME enhancer (*DME-lacZ*, β-Galactosidase immunostaining (green). AbdA variants were ubiquitously expressed with the *arm-Gal4* driver. A representative embryo for each AbdA variant is shown. Boxed areas highlight thoracic segments where the effect of AbdA variants was determined.
(PDF)

Figure S8 (Full data for Figure 7B.) AbdA protein domain requirements for the regulation of the *Antp* target gene. The regulatory effect of AbdA variants on *Antp* expression was determined by Antp immunostainings (green). AbdA variants were ubiquitously expressed with the *arm-Gal4* driver. A representative embryo for each AbdA variant (red) is shown. Boxed areas highlight thoracic segments where the effect of AbdA variants was determined.
(PDF)

Figure S9 (Full data for Figure 7C.) AbdA protein domain requirements for A2 epidermal morphology. Abdominal segments harbour large and refringent denticles, organised in a trapezoid in A2 but not A1, while thoracic segments T2–T3 harbour smaller and less refringent denticles organised in a linear manner. The first thoracic segment T1 in addition harbours a specific feature termed the beard (arrow). Ubiquitous expression of AbdA variants with *arm-Gal4* suppresses the beard and promotes the formation of abdominal like denticle belts to different extent.
(PDF)

Figure S10 (Full data for Figure 7D.) AbdA protein domain requirements for larval locomotion. Upon ubiquitous expression of wild type or AbdA variants through the *arm-Gal4* driver, five forward waves (randomly selected) were scored for ectopic dorso/ventral (D/V) movement in the T3 thoracic segment. The number of D/V movements in T3 during the five scored forward waves is reported for each embryo scored. For *hth*, waves were scored in *hth^P2^* homozygote context.
(PDF)

Text S1 Supporting Materials and Methods
(DOCX)
