# Supplementary figures and images for: Insights into Hox Protein Function from a Large Scale Combinatorial Analysis of Protein Domains
Source: PLoS Genet. 2011 Oct 27;7(10):e1002302. doi: 10.1371/journal.pgen.1002302 (PMC3203194; doi:10.1371/journal.pgen.1002302)

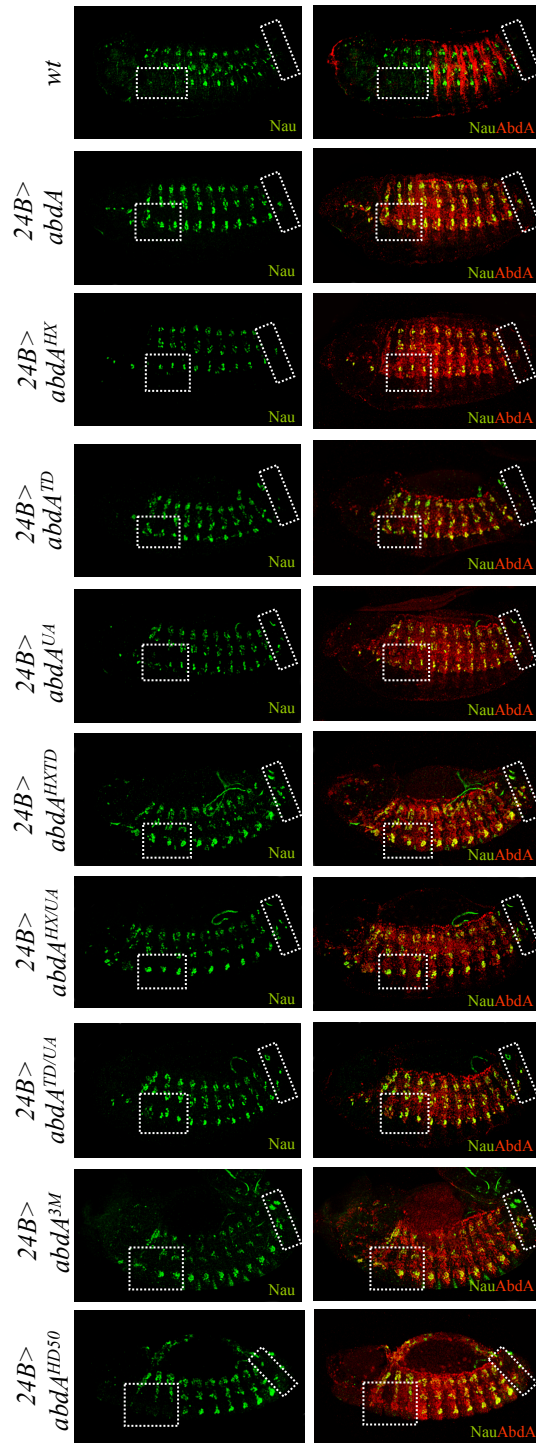

**Figure S1**

Supplement: Figure S1 — (Full data for Figure 2.) AbdA protein domain requirements for somatic muscle specification. Somatic muscle cells are visualized by Nau immunostaining (green). A representative embryo is shown for each AbdA variant, as indicated. AbdA variants were ubiquitously expressed (red) in the mesoderm with the 24B-Gal4 driver. Dotted white rectangles highlights segments (thoracic or abdominal segment A8) where the effect of AbdA variants was determined. (PDF) [file pgen.1002302.s001.pdf]

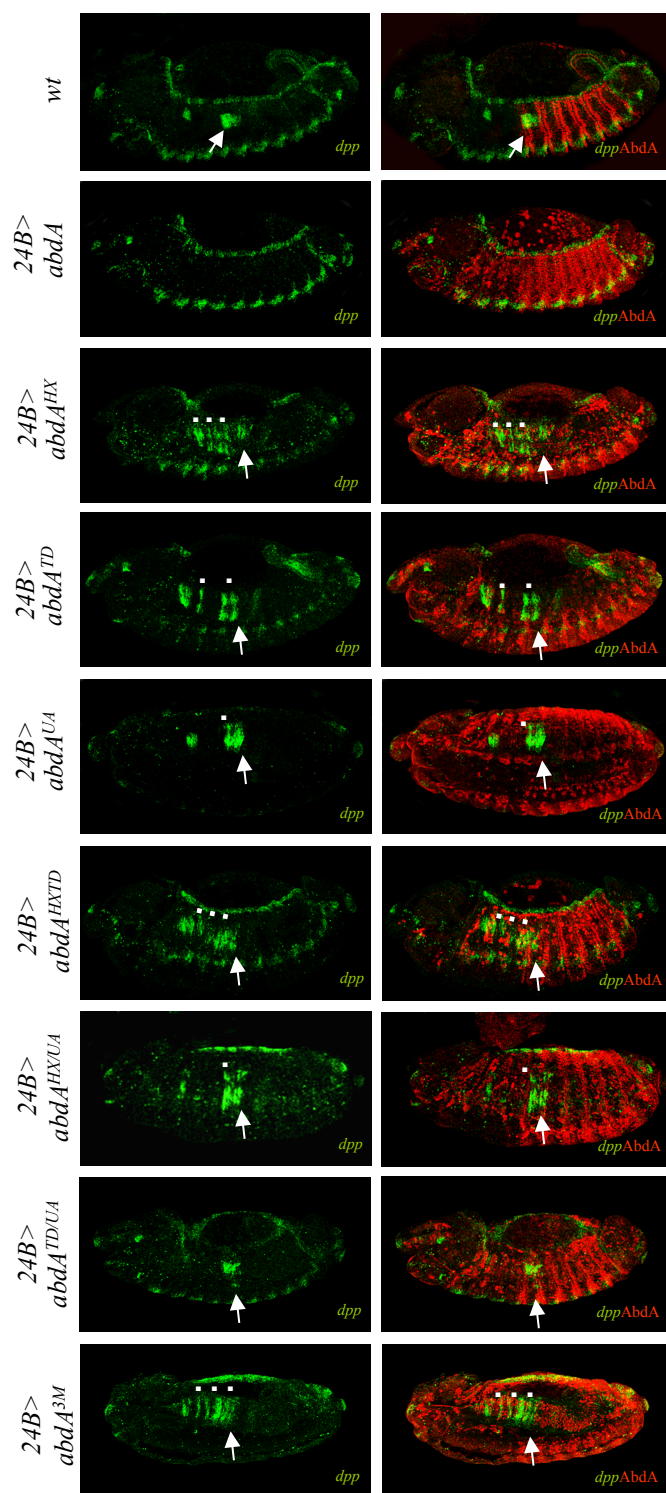

**Figure S2**

Supplement: Figure S2 — (Full data for Figure 4A.) AbdA protein domain requirements for the regulation of the dpp direct target gene. The regulatory effect of AbdA variants on dpp expression was determined by in situ hybridisation to dpp transcripts (green). Arrow indicates the expression of dpp in PS7 of the visceral mesoderm. Gain of dpp expression in the visceral mesoderm is indicated by white dots, while loss of PS7 expression is denoted by the absence of arrow. AbdA variants were ubiquitously expressed (red) in the mesoderm with the 24B-Gal4 driver. A representative embryo is shown for each AbdA variant. (PDF) [file pgen.1002302.s002.pdf]

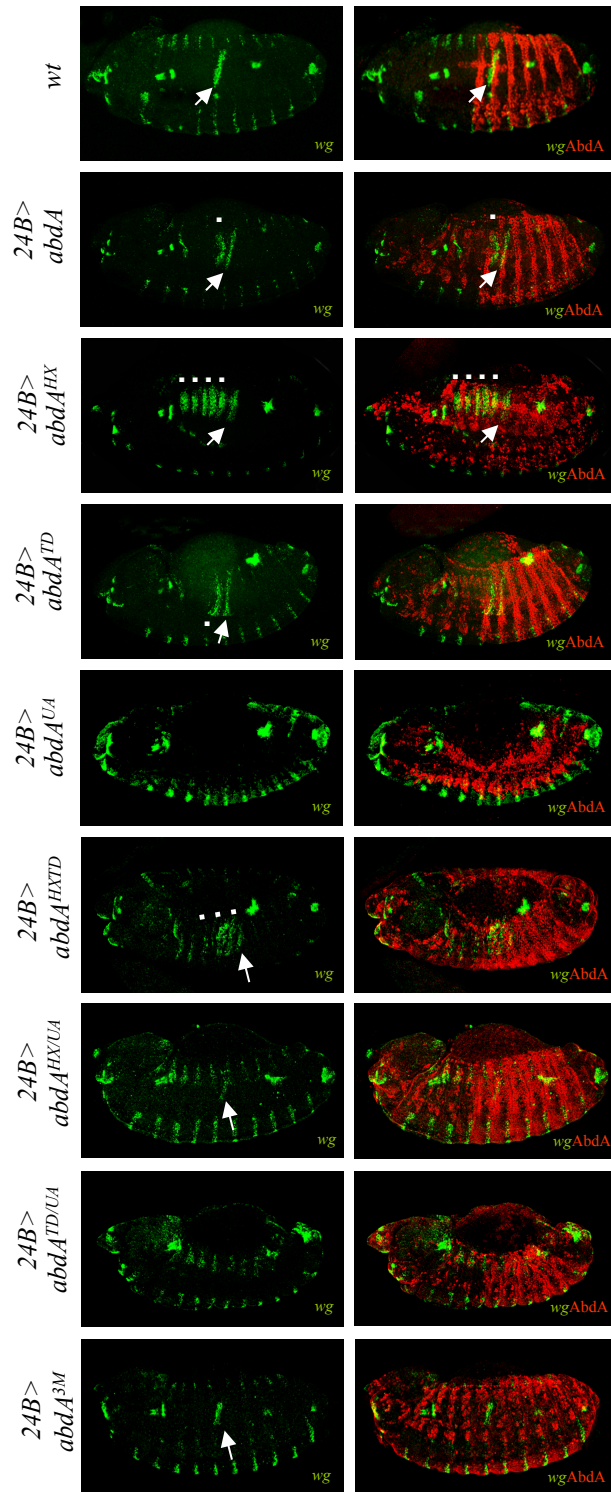

Figure S3

Supplement: Figure S3 — (Full data for Figure 4B.) AbdA protein domain requirements for the regulation of the wg direct target gene. The regulatory effect of AbdA variants on wg expression was determined by in situ hybridisation to wg transcripts (green). Arrow indicates the expression of wg in PS8 of the visceral mesoderm. Gain of wg expression in the visceral mesoderm is indicated by white dots, while loss of PS8 expression is denoted by the absence of arrow. Restricted PS8 activation of wg by AbdA results from the action of the Dpp signal, locally produced by PS7 cells under the control of the Ubx protein [40]. Accordingly, anterior ectopic expression of AbdA only results in a mild activation of wg, as activation only occurs in cells experiencing partial repression of dpp [27]. Previous work showed that the HX mutation results in a protein that activates dpp instead of repressing it, and consequently more efficiently activates wg [41]. (PDF) [file pgen.1002302.s003.pdf]

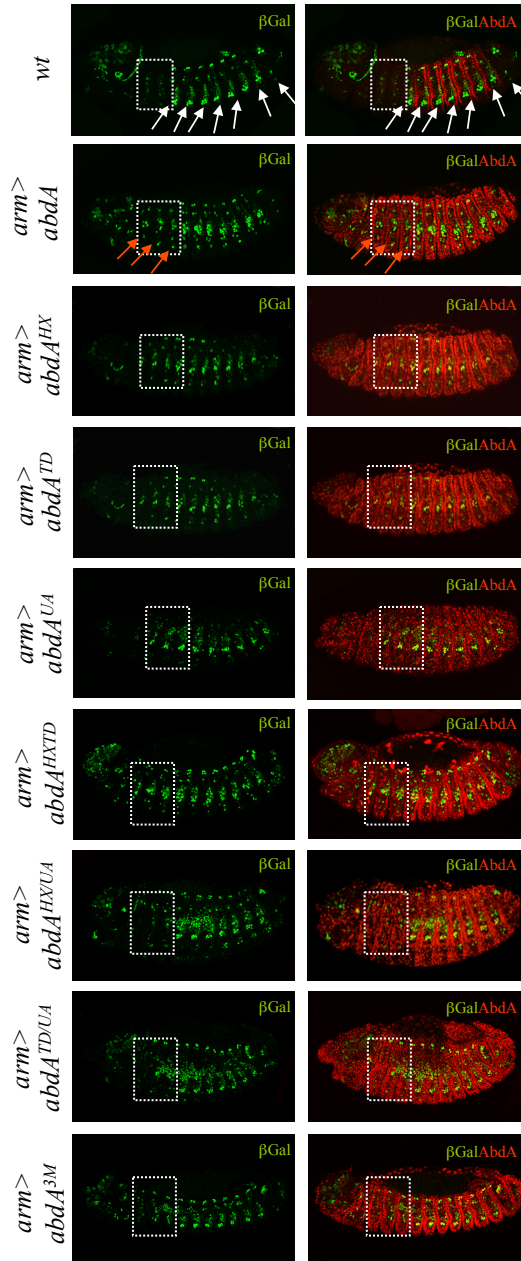

**Figure S4**

Supplement: Figure S4 — (Full data for Figure 5.) AbdA protein domain requirements for oenocytes specification. Oenocytes, restricted to A1–A7 abdominal segments, were marked using a seven-up svp-lacZ construct (β-galactosidase staining in green). Ubiquitous expression of AbdA variants (red) with arm-Gal4 induces the formation of ectopic oenocytes in thoracic segments. A representative embryo is shown for each AbdA variant. Boxed areas highlight thoracic segments. (PDF) [file pgen.1002302.s004.pdf]

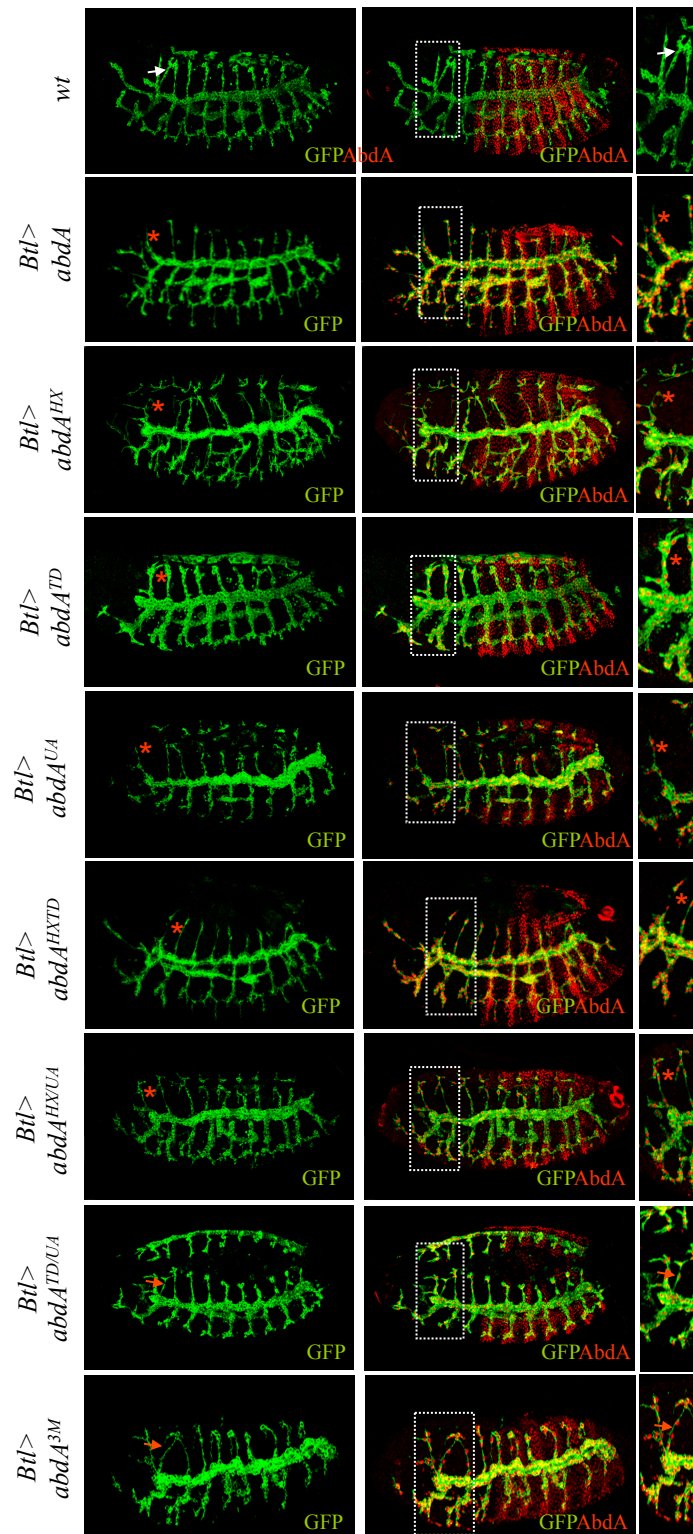

Figure S5

Supplement: Figure S5 — (Full data for Figure 6A.) AbdA protein domain requirements for cerebral branch specification. The breathless btl-Gal4 driver, specific to tracheal branches, was used to simultaneously express the AbdA variants (red) and the GFP reporter protein (green), allowing visualisation of tracheal defects. Presence (white arrow) or absence (red star) of the cerebral branch following ectopic expression of the AbdA variants is shown. (PDF) [file pgen.1002302.s005.pdf]

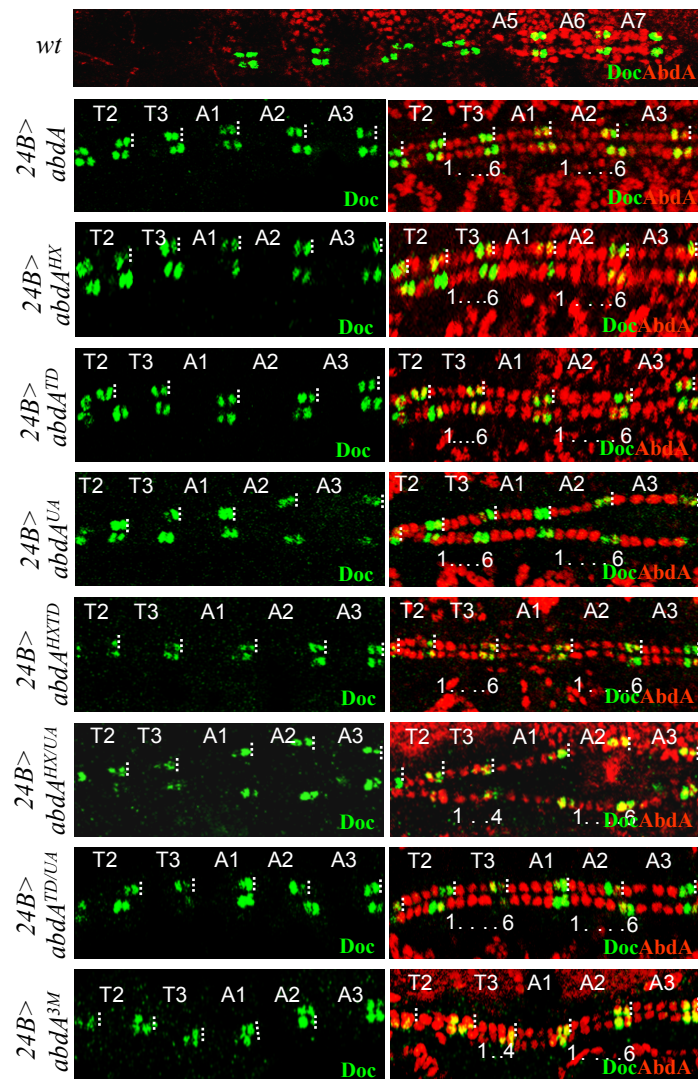

**Figure S6**

Supplement: Figure S6 — (Full data for Figure 6B.) AbdA protein domain requirements for the specification of heart cells. Thoracic segments are formed of four pairs of cardiac cells, while abdominal ones are composed of six pairs of cardiac cells. The two supplementary pairs of abdominal cardiac cells express Doc1 (green). Ectopic expression of AbdA (red) in the mesoderm driven with the 24B-Gal4 driver induces additional Doc1-expressing cells in thoracic segments that are now composed of six pairs of cells. A representative embryo is shown for each AbdA variant. (PDF) [file pgen.1002302.s006.pdf]

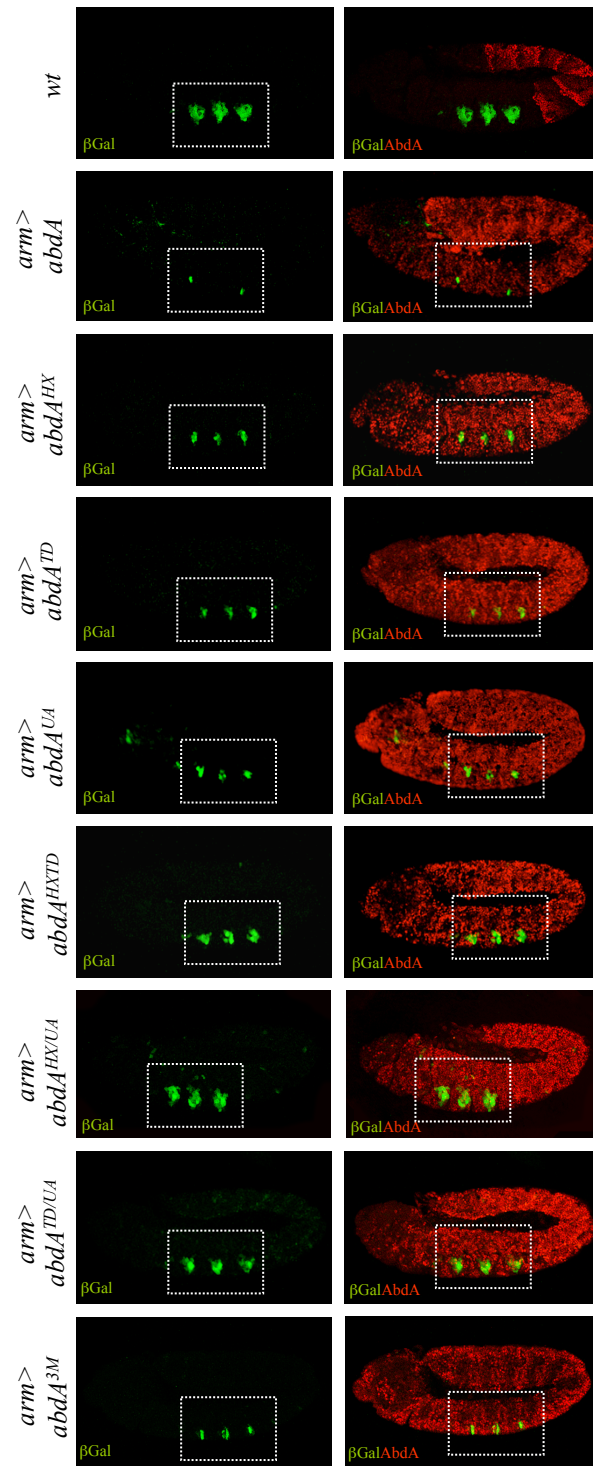

**Figure S7**

Supplement: Figure S7 — (Full data for Figure 7A.) AbdA protein domain requirements for the regulation of the Dll direct target gene. The regulatory effect of AbdA variants (red) on Dll expression was determined by the activity of the Dll DME enhancer (DME-lacZ, β-Galactosidase immunostaining (green). AbdA variants were ubiquitously expressed with the arm-Gal4 driver. A representative embryo for each AbdA variant is shown. Boxed areas highlight thoracic segments where the effect of AbdA variants was determined. (PDF) [file pgen.1002302.s007.pdf]

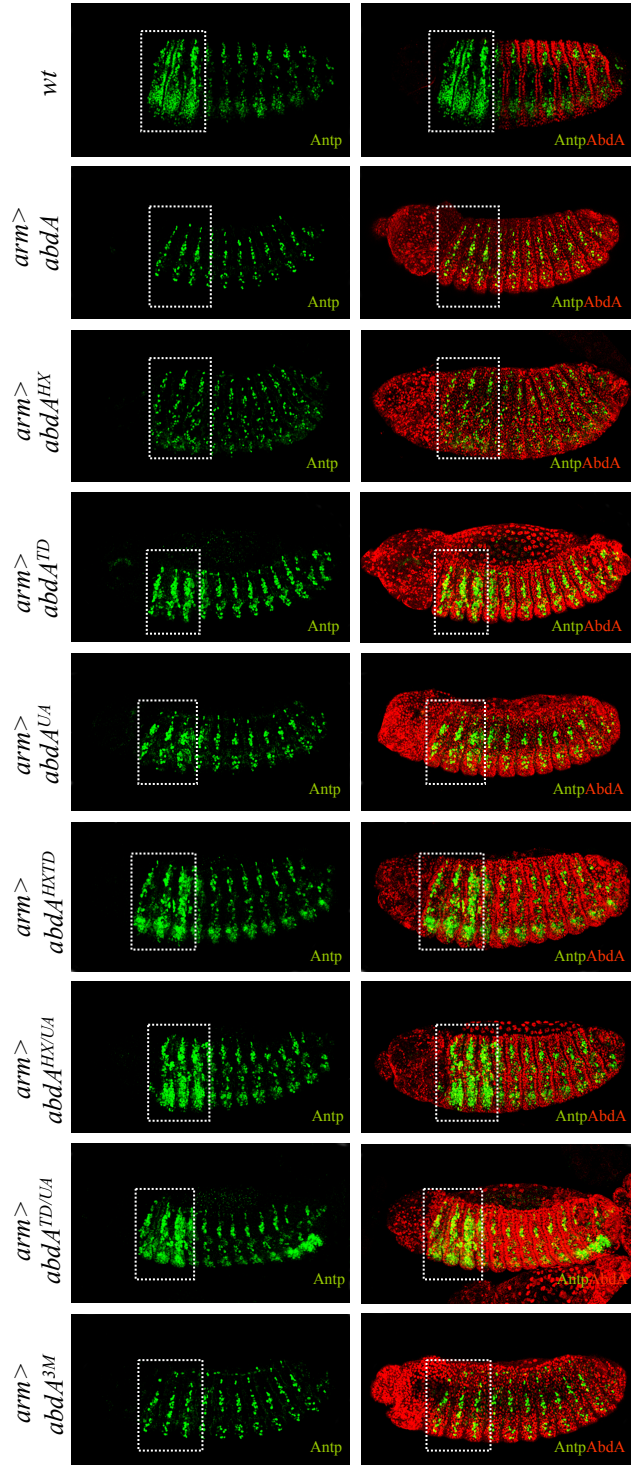

**Figure S8**

Supplement: Figure S8 — (Full data for Figure 7B.) AbdA protein domain requirements for the regulation of the Antp target gene. The regulatory effect of AbdA variants on Antp expression was determined by Antp immunostainings (green). AbdA variants were ubiquitously expressed with the arm-Gal4 driver. A representative embryo for each AbdA variant (red) is shown. Boxed areas highlight thoracic segments where the effect of AbdA variants was determined. (PDF) [file pgen.1002302.s008.pdf]
