# Supplementary material for: Insights into Hox Protein Function from a Large Scale Combinatorial Analysis of Protein Domains
Source: PLoS Genet. 2011 Oct 27;7(10):e1002302. doi: 10.1371/journal.pgen.1002302 (PMC3203194; doi:10.1371/journal.pgen.1002302)
Supplement: Figure S10 — (Full data for Figure 7D.) AbdA protein domain requirements for larval locomotion. Upon ubiquitous expression of wild type or AbdA variants through the arm-Gal4 driver, five forward waves (randomly selected) were scored for ectopic dorso/ventral (D/V) movement in the T3 thoracic segment. The number of D/V movements in T3 during the five scored forward waves is reported for each embryo scored. For hth, waves were scored in hthP2 homozygote context. (PDF) [file pgen.1002302.s010.pdf]

	wt	HX	TD	UA	HX<br>TD	HX<br>UA	TD<br>UA	3M	HTH
embryo 1	5	5	5	3	2	2	1	5	0
embryo 2	4	5	5	3	4	1	0	5	0
embryo 3	5	5	5	5	5	4	2	4	0
embryo 4	1	5	4	5	5	5	3	5	0
embryo 5	5	5	5	3	5	4	3	5	0
embryo 6	5	3	4	3	5	2	1	5	0
embryo 7	5	5	5	4	5	0	5	4	0
embryo 8	5	3	4	5	3	0	3	4	0
embryo 9	5	5	5	0	4	2	5	5	0
embryo 10	5	5	5	4	5	2	2	4	0
**total**	**45**	**46**	**47**	**35**	**43**	**22**	**25**	**46**	**0**

Fig. S10
