# Supplementary material for: Insights into Hox Protein Function from a Large Scale Combinatorial Analysis of Protein Domains
Source: PLoS Genet. 2011 Oct 27;7(10):e1002302. doi: 10.1371/journal.pgen.1002302 (PMC3203194; doi:10.1371/journal.pgen.1002302)
Supplement: Text S1 — Supporting Materials and Methods (DOCX) [file pgen.1002302.s011.docx]

**Supplementary Materials and Methods**

For all assays, embryos were collected at temperatures that allow ectopic expression at levels similar (+/- 15%) to endogenous AbdA in the A2 segment. These conditions depend on the AbdA variant transgenic line as well as the driver used. A detailed description of the procedure used to select conditions allowing physiological levels of expression can be found in [22]. To quantify the activity of AbdA variants, at least ten different embryos for each variant were scored.

***1- Quantification of AbdA repressive activity on the Distalless target enhancer DME.*** Levels of AbdA repressive activity on *DME-lacZ* were deduced from the quantification of the fluorescent staining for lacZ-positive cells in the three thoracic segments. The percentage of surface reduction was deduced by comparison to the wild type *DME* expression using the AxioVision LE4.5 (Zeiss) measurement tool. For example, an AbdA variant leading to a mean surface reduction of 45% was considered as having a repressive activity of 55%.

***2- Quantification of AbdA repressive activity on the Antennapedia (Antp) target gene.*** Levels of Hox repressive activities on *Antp* were deduced from the quantification of the reduction of the fluorescent staining against Antp in the epidermis of thoracic segments. The percentage of surface reduction was deduced from comparison to the wild type Antp expression by using the AxioVision LE4.5 (Zeiss) measurement tool.

***3- Quantification of AbdA regulatory activity on the wingless (wg) target gene.***

Levels of AbdA regulatory activity on *wg* were deduced from the quantification of fluorescent *in situ* hybridization against *wg.* Measures were performed in the visceral mesoderm of stage 14 embryos. Arbitrary values have been assigned to regulatory activities of AbdA variants, depending of the nature of the *wg* signal: no effect (0%) was considered when ectopic expression of AbdA variants did not change the expression profile of *wg* in the parasegment (PS) 8; activation activities vary from 16,5% (one supplementary anterior *wg* signal in addition to the *wg* signal in PS8) to 33% (two supplementary anterior *wg* signals), 49,5% (three supplementary anterior *wg* signals), 66% (four supplementary anterior *wg* signals), 82,5% (five supplementary anterior *wg* signals), and 99% (six supplementary anterior *wg* signals); minus 100% was arbitrary assigned to AbdA variants leading to a loss of *wg* expression in PS8.

***4- Quantification of AbdA regulatory activity on the decapentaplegic (dpp) target gene.***

Levels of regulatory activities were deduced from the quantification of fluorescent *in situ* hybridization against *dpp*. Measures were performed in the visceral mesoderm of stage 14 embryos. Arbitrary values have been assigned to regulatory activities of Hox variants on *dpp*:

0% was assigned when ectopic expression of Hox variants did not change the expression profile of *dpp* in PS7; 100 % was assigned to full repression of *dpp* in PS7. Negative values were assigned for *dpp* activation in the visceral mesoderm as follows: -16,5% (one supplementary *dpp* signal in addition to *dpp* signal in PS7); -33% (two supplementary *dpp* signals); -50% (three supplementary *dpp* signals), -66% (four supplementary *dpp* signals), -82,5% (five supplementary *dpp* signals), and -100% (six supplementary *dpp* signals).

***5- Quantification of AbdA potential to specify abdmominal specific somatic muscle cells.***

Levels of AbdA regulatory activities on abdominal specific somatic muscle cells were deduced from the quantification of fluorescent staining against Nautilus (Nau): Nau is expressed in muscle precursor cells, including abdominal specific ventral clusters in segments A1 to A7. Arbitrary values were assigned to AbdA variants, depending of their effect on Nau expression: 25% of activation activity correspond to ectopic expression of Nau in one supplementary segment, either in thorax (T3) or in abdomen (A8), 50% to ectopic activation of Nau in two supplementary segments, 75% to ectopic activation of Nau in three supplementary segments, and 100% to ectopic activation of Nau in four supplementary segments (T1 to T3, and A8).

***6- Quantification of AbdA repressive activity on the NB5-6 cell lineage.***

Levels of AbdA repressive activity on the NB5-6 cell lineage were deduced from the *lbe(K)-Gal4* driven GFP reporter activity*.* Average number of NB5-6 cells in wild type thoracic and abdominal segments was previously estimated at 16 and 6 cells respectively [39]. These values were considered as references for full (100%) or complete loss (0%) of repressive activities of AbdA variants on NB5-6 lineage. Intermediate repressive levels were deduced from the quantification of NB5-6 cells number in T3 as following: 10% (15 remaining NB5-6 cells in T3), 20% (14 remaining NB5-6 cells in T3), 30% (13 remaining NB5-6 cells in T3), 40% (12 remaining NB5-6 cells in T3), 50% (11 remaining NB5-6 cells in T3), 60% (10 remaining NB5-6 cells in T3), 70% (9 remaining NB5-6 cells in T3), 80% (8 remaining NB5-6 cells in T3), 90% (7 remaining NB5-6 cells in T3).

***7- Quantification of AbdA repressive activity on cerebral branch (CB) formation in the T2 segment.***

Levels of repressive activity on CB formation was followed by a *btl-Gal4*-driven *UAS-GFP* reporter. Only full repression of CB formation was considered and repressive activities of AbdA variants thus correspond to either 0% (no repression of CB formation) or 100% (full repression of CB formation).

***8- Quantification of AbdA potential to specify a six-cell lineage in the heart****.*

Levels of six cell lineage inducing capacity was scored in T2 and T3 following Doc1 staining. Induction from 8 to 12 cells in T2 and T3 was considered as 100%; Induction from 8 to 11 cells was considered as 75%; Induction from 8 to 10 cells was considered as 50%; Induction from 8 to 9 cells was considered as 25%; no Induction (8 cells in T2 and T3) corresponded to 0%.

***9- Quantification of AbdA oenocyte inducing activity****.*

AbdA oenocyte inducing potential (ectopic thoracic *svp-lacZ* activity*)* was deduced from the number of thoracic segments containing ectopic oenocytes upon ubiquitous AbdA variant expression: 100% was assigned to a protein inducing ectopic oenocytes in the three thoracic segments; 67% and 33% were assigned to AbdA variants inducing ectopic oenocytes in two or one thoracic segments, respectively; 0% was assigned to a protein that is unable to induce any oenocytes in thoracic segments. Of note, ectopic oenocytes were never observed in the posterior A8 segment.

***10- Quantification of AbdA potential to specify A2 epidermal morphology****.*

AbdA potential to specify A2 epidermal morphology was defined by assigning values as follows: 100% for complete transformation of thoracic segments towards A2 morphology, a transformation promoted by wild type AbdA (transformation of denticle identity from thoracic to abdominal type, trapezoidal shape organisation of the denticle belt, full suppression of the beard); 75% for weak transformations towards A2 morphology (transformation of denticle identity from thoracic to abdominal type, incomplete trapezoidal shape organisation of denticle belts, full suppression of the beard); 50% for transformations towards A1-like morphology (transformation of denticle identity from thoracic to abdominal type, linear organisation of denticle similar to that found in A1, full suppression of the beard); 25 % for weak transformations towards A1 morphology (transformation of denticle identity from thoracic to abdominal type, linear organisation of denticle similar to that found in A1, partial suppression of the beard). 0% for no transformation.

***11- Quantification of AbdA regulatory activity in controlling larval locomotion****.*

Upon ubiquitous expression of AbdA variants, five forward waves (randomly selected) were scored for ectopic dorso/ventral (D/V) movement in the T3 thoracic segment. The total number of D/V movements in T3 during the five scored forward waves was arbitrary converted as a percentage of Hox activity: from 100% (5 D/V movements in T3 during the five scored forward waves) to 80 % (four D/V movements in T3 during the five scored forward waves), 60 % (three D/V movements in T3 during the five scored forward waves), 40% (two D/V movements in T3 during the five scored forward waves), 20% (one D/V movement in T3 during the five scored forward waves) and 0% (zero D/V movement in T3 during the five scored forward waves).
