# Supplementary material for: Insights into Hox Protein Function from a Large Scale Combinatorial Analysis of Protein Domains
Source: PLoS Genet. 2011 Oct 27;7(10):e1002302. doi: 10.1371/journal.pgen.1002302 (PMC3203194; doi:10.1371/journal.pgen.1002302)
Supplement: Figure S9 — (Full data for Figure 7C.) AbdA protein domain requirements for A2 epidermal morphology. Abdominal segments harbour large and refringent denticles, organised in a trapezoid in A2 but not A1, while thoracic segments T2–T3 harbour smaller and less refringent denticles organised in a linear manner. The first thoracic segment T1 in addition harbours a specific feature termed the beard (arrow). Ubiquitous expression of AbdA variants with arm-Gal4 suppresses the beard and promotes the formation of abdominal like denticle belts to different extent. (PDF) [file pgen.1002302.s009.pdf]

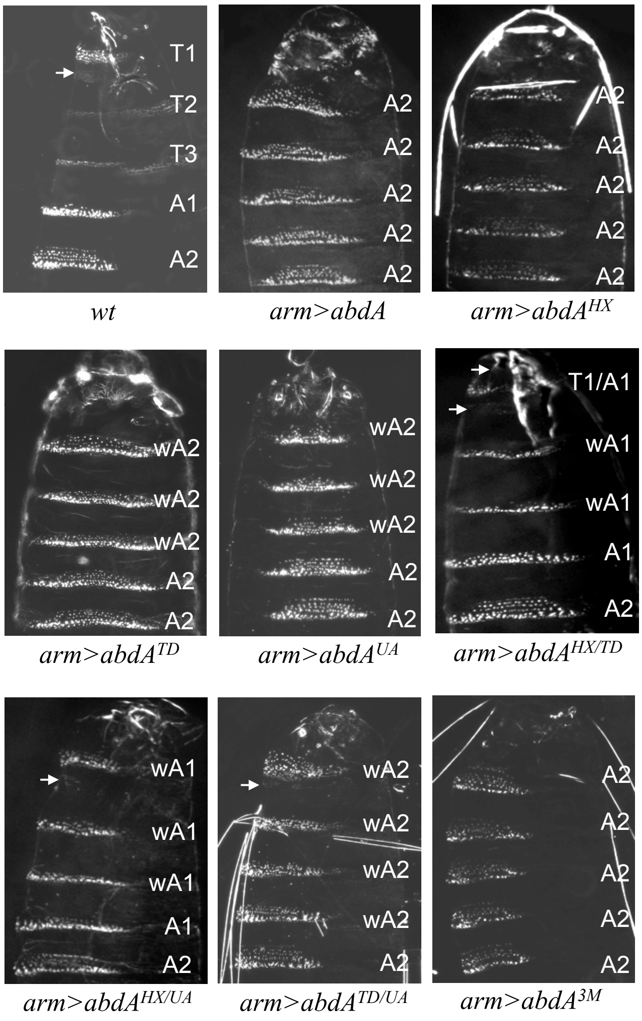
T1
T2
T3
A1
A2
wt
A2
A2
A2
A2
A2
arm>abdA
A2
A2
A2
A2
A2
arm>abdA^HX
wA2
wA2
wA2
A2
A2
arm>abdA^TD
wA2
wA2
wA2
A2
A2
arm>abdA^UA
T1/A1
wA1
wA1
A1
A2
arm>abdA^HX/TD
wA1
wA1
wA1
A1
A2
arm>abdA^HX/UA
wA2
wA2
wA2
wA2
A2
arm>abdA^TD/UA
A2
A2
A2
A2
A2
arm>abdA^3M

Figure S9
